# Supplementary material for: ARIH1 activates STING-mediated T-cell activation and sensitizes tumors to immune checkpoint blockade
Source: Nat Commun. 2023 Jul 10;14:4066. doi: 10.1038/s41467-023-39920-5 (PMC10333294; doi:10.1038/s41467-023-39920-5)
Supplement: Supplementary file 1 — Supplementary Information [file 41467_2023_39920_MOESM1_ESM.pdf]

ARIH1 activates STING-mediated T-cell activation and sensitizes tumors to immune checkpoint blockade

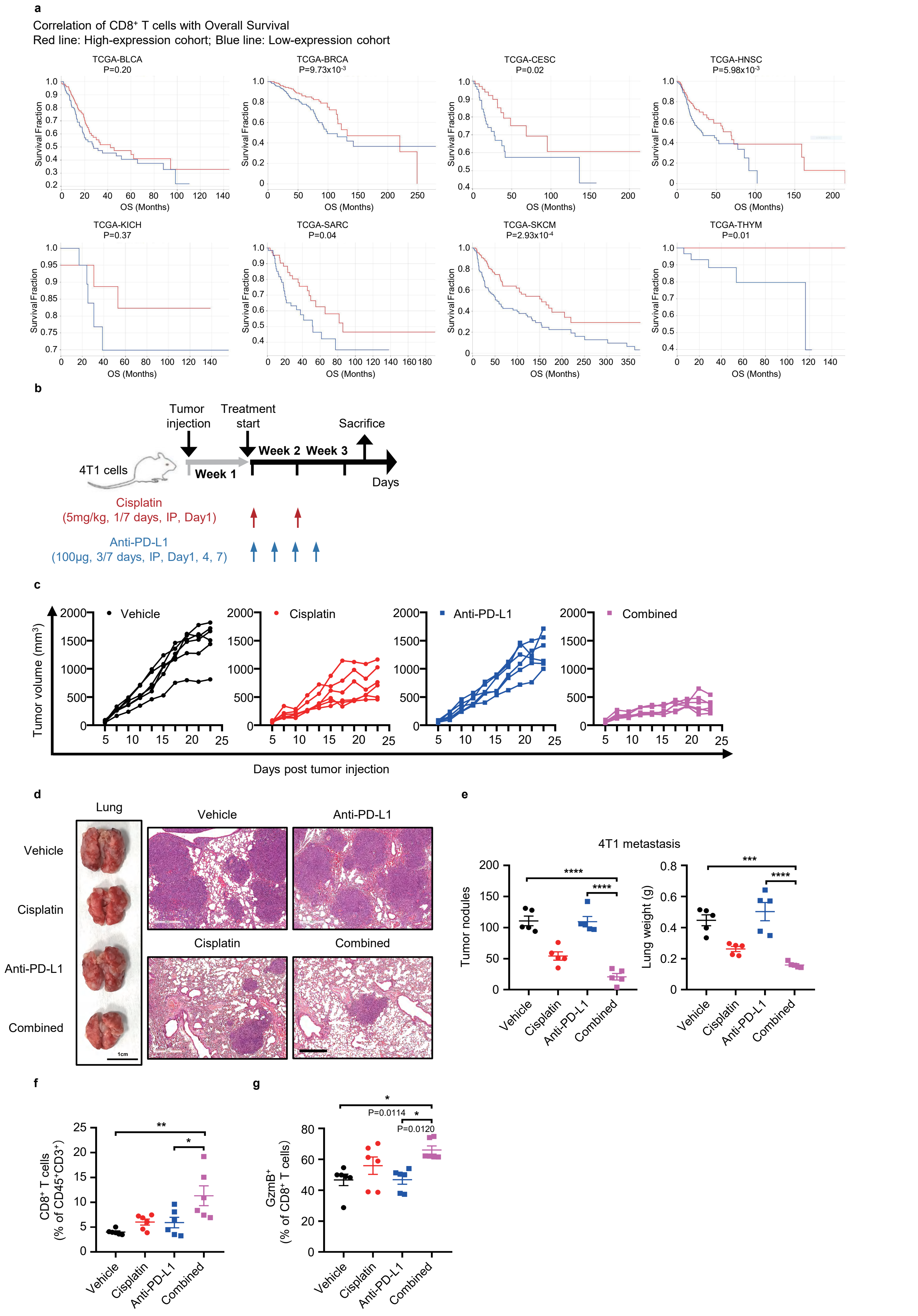

a
Correlation of CD8+ T cells with Overall Survival
Red line: High-expression cohort; Blue line: Low-expression cohort
TCGA-BLCA P=0.20
TCGA-BRCA P=9.73x10-3
TCGA-CESC P=0.02
TCGA-HNSC P=5.98x10-3
TCGA-KICH P=0.37
TCGA-SARC P=0.04
TCGA-SKCM P=2.93x10-4
TCGA-THYM P=0.01
Survival Fraction
OS (Months)
b
Tumor injection
Treatment start
Sacrifice
4T1 cells
Week 1
Week 2
Week 3
Days
Cisplatin (5mg/kg, 1/7 days, IP, Day1)
Anti-PD-L1 (100μg, 3/7 days, IP, Day1, 4, 7)
c
Vehicle
Cisplatin
Anti-PD-L1
Combined
Tumor volume (mm3)
Days post tumor injection
d
Lung
Vehicle
Cisplatin
Anti-PD-L1
Combined
1cm
e
4T1 metastasis
Tumor nodules
Lung weight (g)
****
***
f
CD8+ T cells (% of CD45+CD3+)
**
*
g
GzmB+ (% of CD8+ T cells)
*
P=0.0114
P=0.0120

Supplementary Figure 1: Cisplatin enhances the efficacy of anti-PD-L1 antibody.
a. Association of $CD8^+$ T cells with overall survival (OS) in BLCA (Bladder Urothelial Carcinoma, High (n=303), Low (n=101)), BRCA (Breast Invasive Carcinoma, High (n=814), Low (n=271)), CESC (Cervical Squamous Cell Carcinoma and Endocervical Adenocarcinoma, High (n=230), Low (n=76)), HNSC (Head and Neck Squamous Cell Carcinoma, High (n=389), Low (n=130)), KICH (Kidney Chromophobe, High (n=50), Low (n=16)), SARC (Sarcoma, High (n=197), Low (n=65)), SKCM (Skin Cutaneous Melanoma, High (n=346), Low (n=115)) and THYM (Thymoma, High (n=89), Low (n=29)) from the Gene Expression Profiling Interactive Analysis platform (http://gepia2021.cancer-pku.cn/survival.html). Log-rank test. **b.** A schematic model that illustrates the treatment plan for mice bearing tumors in the female BALB/c mice (n = 6 per group, 6-8 week old) treated with vehicle, cisplatin alone (5mg/kg), anti-PD-L1 alone (100μg) and cisplatin+anti-PD-L1 for a total 2-week treatment course after subcutaneous injection of 5×10^5 4T1 cells.This image was created by the first author. **c.** Tumor growth curves for each tumor bearing BALB/c mouse (n=6 per group) in Figure 1**a**. **d-e.** Representative images (**d**) and quantification (**e**) of tumor nodules and lung weights of mice at day 16 with the indicated treatments after intravenous injection of 1×10^5 4T1 cells. Scale bar, 400μm. n=5 per group. Data represent means ± SEM, ***P < 0.001 (P = 0.0002), ****P < 0.0001. **f-g.** Flow cytometry analysis for the tumor levels of $CD8^+$ T cells (**f**) and $CD8^+GzmB^+$ T cells (**g**) of the mice as in Figure 1**a**. n=6 mice/group. Data represent means ± SEM. **f** *P < 0.05 (P = 0.0193), **P < 0.01 (P = 0.0015). **g.** *P < 0.05. For **e-g** data, One-way ANOVA test. Source data are provided as a Source Data file.

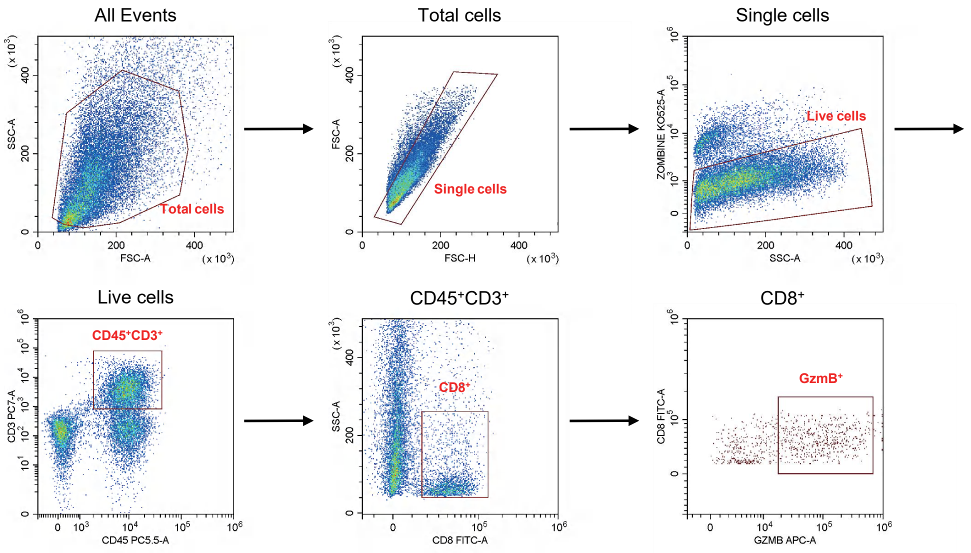

Supplementary Figure 2: Gating procedure for tumor-infiltrating $CD8^+$ T cells and $GzmB^+$ T cells in tumor microenvironment.
This strategy was used for all FACS data panels.

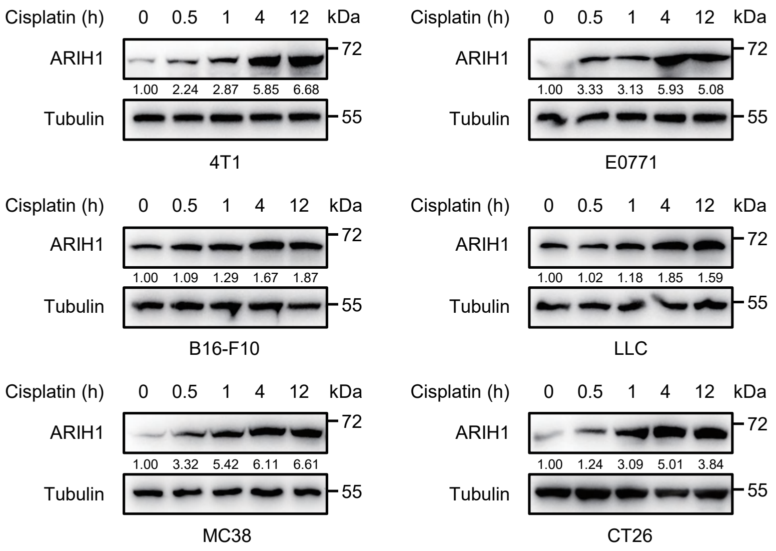

Supplementary Figure 3: Cisplatin increases ARIH1 protein levels.
Immunoblot analysis of ARIH1 levels in tumor cells after treatment with 10 μM cisplatin for indicated times. The numbers under the blots represent the gray scale quantification (ARIH1/Tubulin). Data shown is representative of three independent experiments. Source data are provided as a Source Data file.

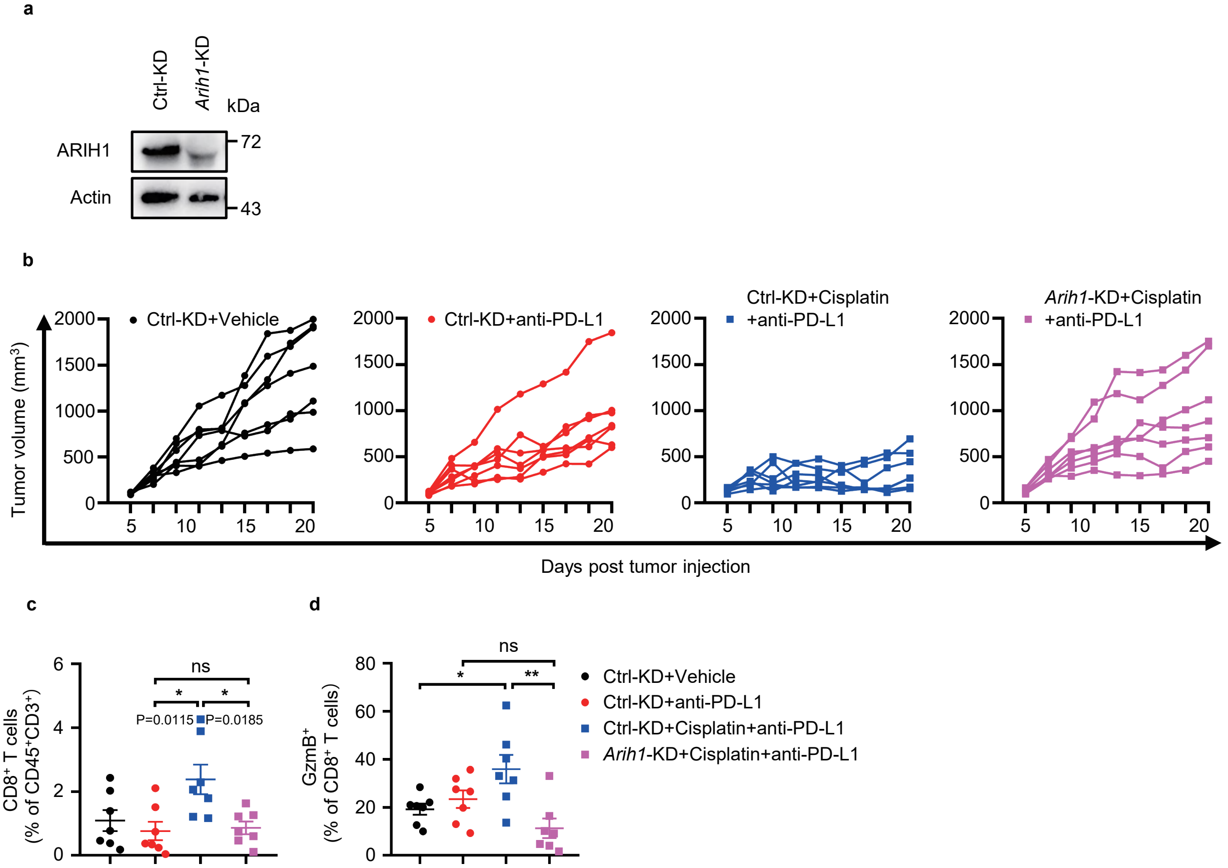

Supplementary Figure 4: ARIH1 knockdown reverses the anti-tumor effect of PD-L1 plus Cisplatin.
a. Immunoblot analysis of ARIH1 levels in Ctrl-KD and *Arih1*-KD 4T1 cells. **b.** Tumor growth in each tumor bearing BALB/c mouse of Ctrl-KD and *Arih1*-KD 4T1 cells with indicated treatments. n=7 mice/group. **c-d.** Quantification of FACS data for tumor infiltrating $CD8^+$ T cells (**c**) and $GzmB^+CD8^+$ T cells (**d**) of mice as in Figure 1**h**. n=7 mice/group. Data represent means ± SEM, **d** $^*P < 0.05$ ($P = 0.0454$), $^{**}P < 0.01$ ($P = 0.0020$), ns, not significant. For **c-d** data, One-way ANOVA test. Data shown in **a** is representative of three independent experiments. Source data are provided as a Source Data file.

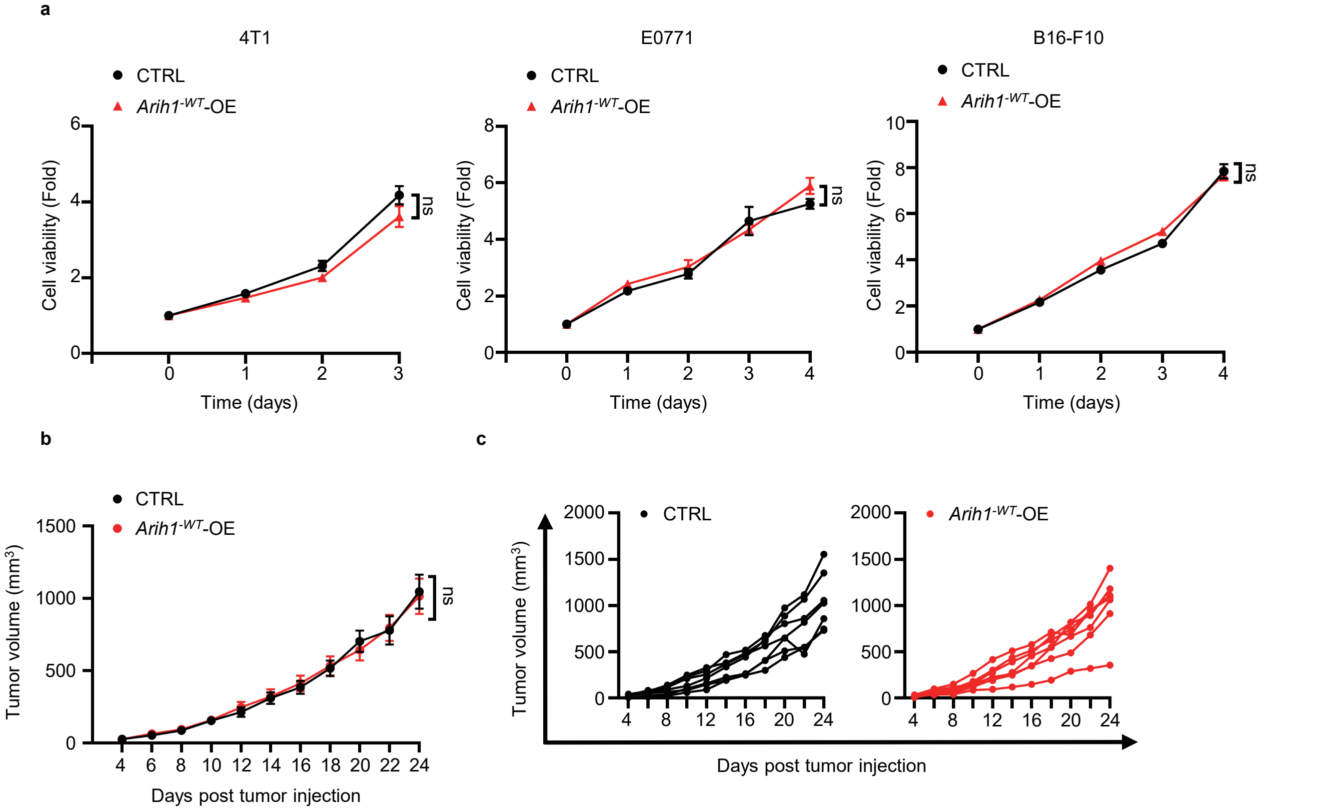

Supplementary Figure 5: Overexpression of ARIH1 shows no effect on the proliferation of tumor cells *in vitro* and in immuno-compromised mice.

a. Tumor cells were infected with an empty vector (CTRL) or *Arih1* overexpressing lentiviral preparations (*Arih1*$^{-WT}$-OE). Cell viability was monitored at indicated time points by an ATP assay. n=5/group. **b-c.**Tumor growth of CTRL and *Arih1*$^{-WT}$-OE 4T1 cells in female nude mice (6-8 week old). n=7 mice/group. For **a-b** data are presented as means ± SEM, Two-way ANOVA test. ns, not significant. Source data are provided as a Source Data file.

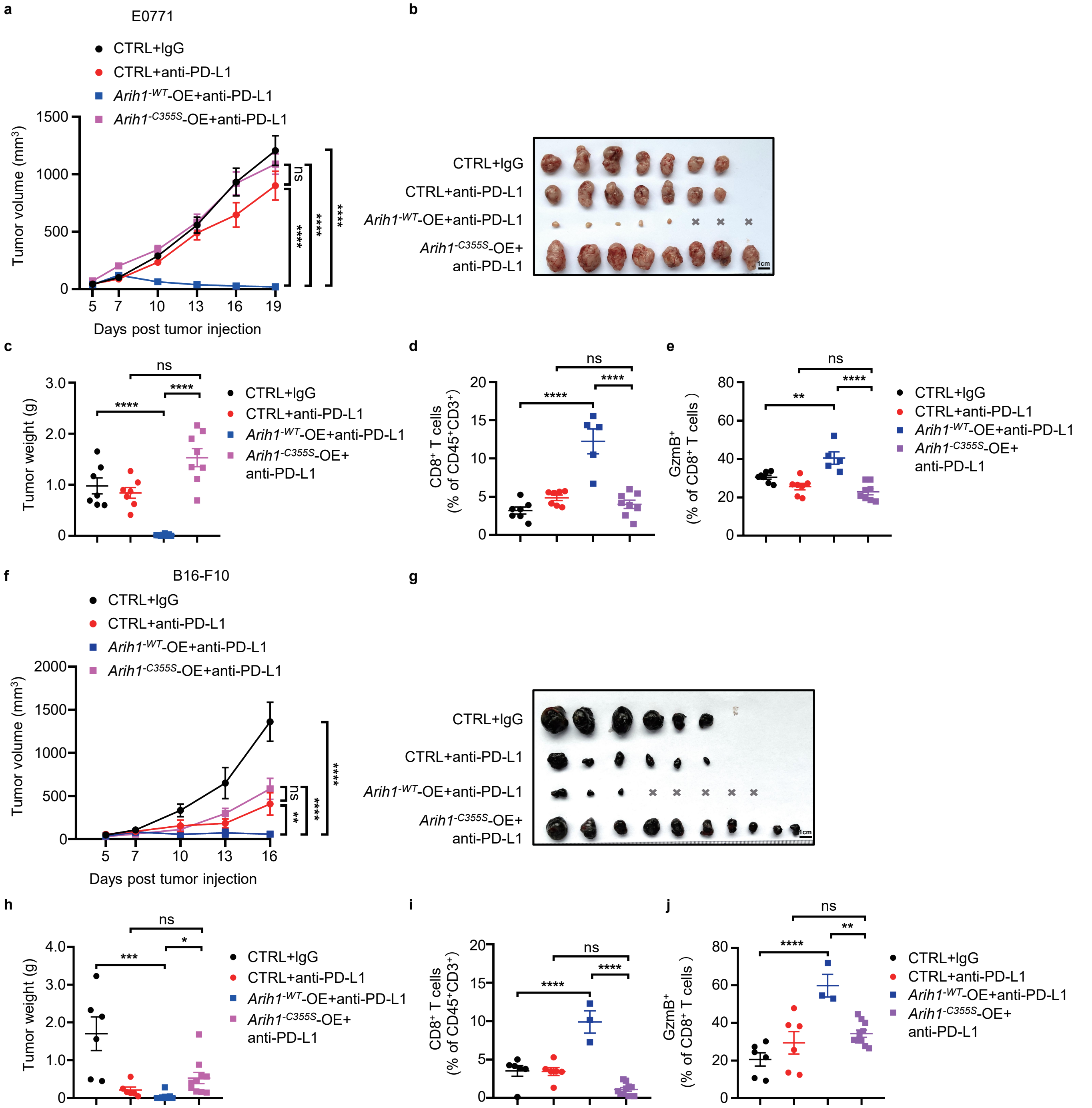

Supplementary Figure 6: The E3 ligase activity is required for ARIH1 enhancing PD-L1 blockade-induced anti-tumor immunity.

a. Tumor growth curves of CTRL, *Arih1*$^{-WT}$-OE, and *Arih1*$^{-C355S}$-OE E0771 cells (8×10^5) in female C57BL/6 mice (n=7, 7, 8, 8 per group, 6-8 week old) with indicated treatments. Data represent means ± SEM, ****P < 0.0001, ns, not significant. **b-c.** Representative image of tumors (**b**) and tumor weights (**c**) of the mice as in (**a**) at Day20 with the indicated treatments. n=7, 7, 8, 8 mice/group. Data represent means ± SEM, ****P < 0.0001, ns, not significant. **d-e.** Quantification of tumor-infiltrating CD8$^+$ T cells (**d**) and GzmB$^+$CD8$^+$ T cells (**e**) of the mice as in (**a**). n=7, 7, 5, 8 mice/group. Data represent means ± SEM, **P < 0.01 (P = 0.0072), ****P < 0.0001, ns, not significant. **f.** Tumor growth curves of CTRL, *Arih1*$^{-WT}$-OE, and *Arih1*$^{-C355S}$-OE B16-F10 cells (5×10^5) in male C57BL/6 mice (n=6, 6, 8, 10 per group, 6-8 week old) with indicated treatments. Data represent means ± SEM, **P < 0.01 (P = 0.0061), ****P < 0.0001, ns, not significant. **g-h.** Representative image of tumors (**g**) and tumor weights (**h**) of the mice as in (**f**) at Day17 with the indicated treatments. n=6, 6, 8, 10 mice/group. Data represent means ± SEM, *P < 0.05 (P = 0.0110), ***P < 0.001 (P = 0.0010), ns, not significant. **i-j.** Quantification of tumor-infiltrating CD8$^+$ T cells (**i**) and GzmB$^+$CD8$^+$ T cells (**j**) of the mice as in (**f**). n=6, 6, 3, 10 mice/group. Data represent means ± SEM, **P < 0.01 (P = 0.0037), ****P < 0.0001, ns, not significant. For **a** and **f** data, Two-way ANOVA test. For **c-e** and **i-j** data, One-way ANOVA test. For **h** data, Two-tailed t-test. Source data are provided as a Source Data file.

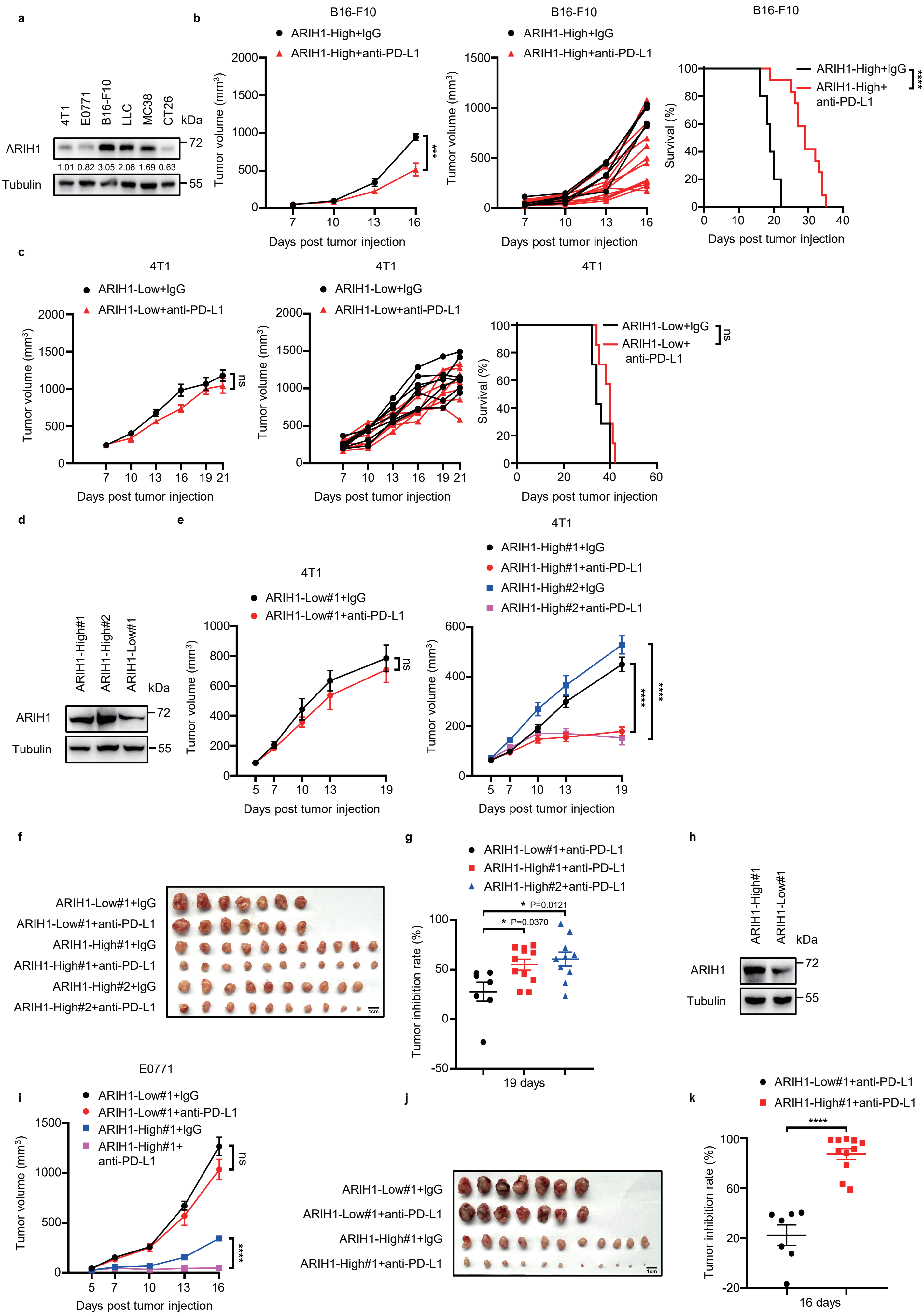

a
4T1
E0771
B16-F10
LLC
MC38
CT26
kDa
ARIH1
72
1.01 0.82 3.05 2.06 1.69 0.63
Tubulin
55
b
B16-F10
ARIH1-High+IgG
ARIH1-High+anti-PD-L1
Tumor volume (mm³)
Days post tumor injection
***
Survival (%)
****
ARIH1-High+ anti-PD-L1
c
4T1
ARIH1-Low+IgG
ARIH1-Low+anti-PD-L1
ARIH1-Low+ anti-PD-L1
ns
d
ARIH1-High#1
ARIH1-High#2
ARIH1-Low#1
e
ARIH1-Low#1+IgG
ARIH1-Low#1+anti-PD-L1
ARIH1-High#1+IgG
ARIH1-High#1+anti-PD-L1
ARIH1-High#2+IgG
ARIH1-High#2+anti-PD-L1
f
1cm
g
Tumor inhibition rate (%)
* P=0.0121
* P=0.0370
19 days
h
i
E0771
j
k
16 days

Supplementary Figure 7: Analysis of ARIH1 levels for immune checkpoint blockade therapy in mouse models.

a. Immunoblot (IB) analysis of ARIH1 levels in tumor cells. The numbers under the blots represent the gray scale quantification (ARIH1/Tubulin). **b.** Tumor growth curves (left and middle) and survival curves (right) of B16-F10 cells (2.5×10^5) in male C57BL/6 mice (n=5, 12 per group, 6-8 week old) with indicated treatments. Data represent means ± SEM, ***P < 0.001 (P = 0.0006). **c.** Tumor growth curves (left and middle) and survival curves (right) of 4T1 cells (5×10^5) in female BALB/c mice (n=7 per group, 6-8 week old) with indicated treatments. **d.** IB analysis of ARIH1 levels in ARIH1-Low and ARIH1-High 4T1 cells. **e.** Tumor growth curves in 5×10^5 4T1 cells with naturelly expressing ARIH1-Low (left) and ARIH1-High (right) in female BALB/c mice (n=7, 7, 11, 11, 10, 10 per group, 6-8 week old) with indicated treatments. Data represent means ± SEM, ****P < 0.0001, ns, not significant. **f.** Tumor image of mice as in (**e**). n=7, 7, 11, 11, 10, 10 mice/group. **g.** Tumor weights inhibition rate in BALB/c mice with ARIH1-Low and ARIH1-High 4T1 cells after indicated treatments at Day19. n=7, 11, 10 mice/group. Data represent means ± SEM, *P < 0.05. **h.** IB analysis of ARIH1 levels in ARIH1-Low and ARIH1-High E0771 cells. **i-j.** Tumor growth curves (**i**) and tumor image (**j**) of E0771(8×10^5) cells with naturelly expressing ARIH1-Low and ARIH1-High in female C57BL/6 mice (n=7, 7, 11, 11 per group, 6-8 week old) with indicated treatments. Data represent means ± SEM, ****P < 0.0001, ns, not significant. **k.** Tumor weights inhibition rate in C57BL/6 mice with ARIH1-Low and ARIH1-High E0771 cells after indicated treatments at Day16. n=7, 11 mice/group. Data represent means ± SEM, ****P < 0.0001. Two-way ANOVA test was used to determine statistical significance for time points when all mice were viable for tumor measurement. Log-rank (Mantel-Cox) test was used to determine the statistical significance for the survival of mice. For **g** data, One-way ANOVA test. For **k** data, Two-tailed t-test. Data shown in **a**, **d**, and **h** are representative of three independent experiments. Source data are provided as a Source Data file.

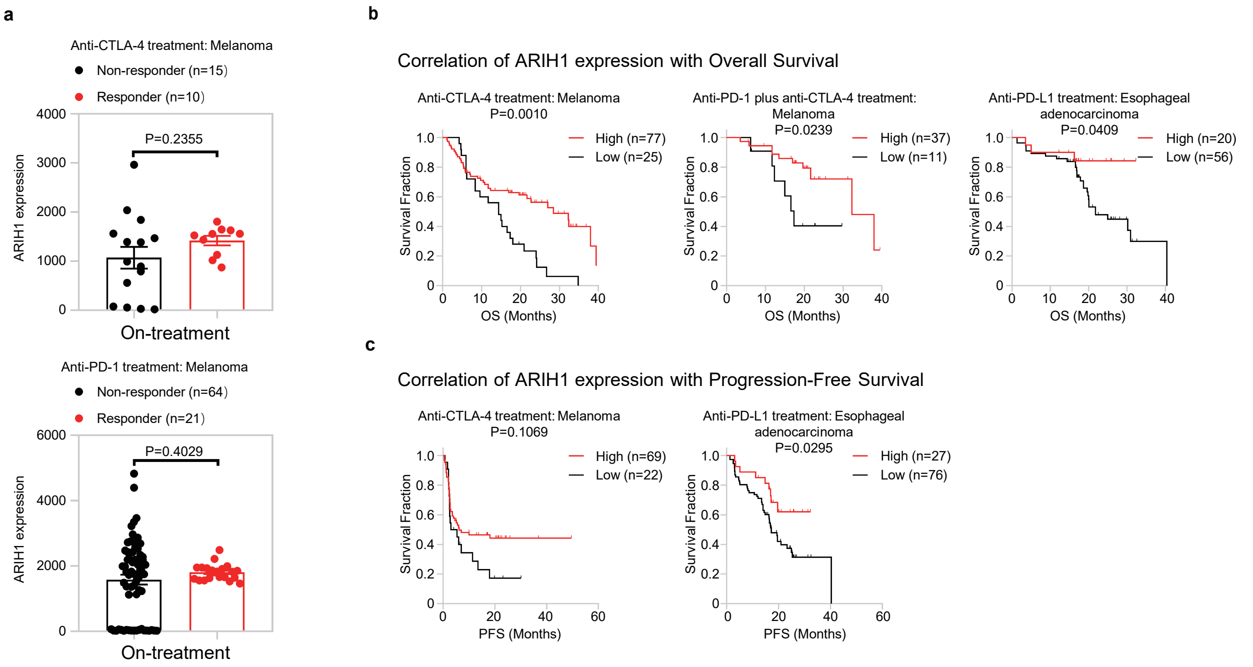

Supplementary Figure 8: Analysis of ARIH1 gene expression for immune checkpoint blockade response in clinical trials.

a. ARIH1 gene levels in indicated responder and non-responder for patients with melanoma enrolled in clinical trials evaluating anti-PD-1/CTLA-4 monotherapy at on-treatment time points from the ROC plotter platform (https://www.rocplot.org/immune). Data is presented as mean ± SEM, Two-tailed t-test. **b-c.** Association of ARIH1 mRNA levels with overall survival (OS) and progression-free survival (PFS) in patients with melanoma or esophageal adenocarcinoma who were enrolled in clinical trials receiving anti-CTLA-4, anti-PD-L1 or the combination of anti-CTLA-4 plus anti-PD-1 therapies from the Kaplan-Meier Plotter platform (https://kmplot.com/analysis/index.php? p=service&cancer =immunotherapy). Log-rank test. Source data are provided as a Source Data file.

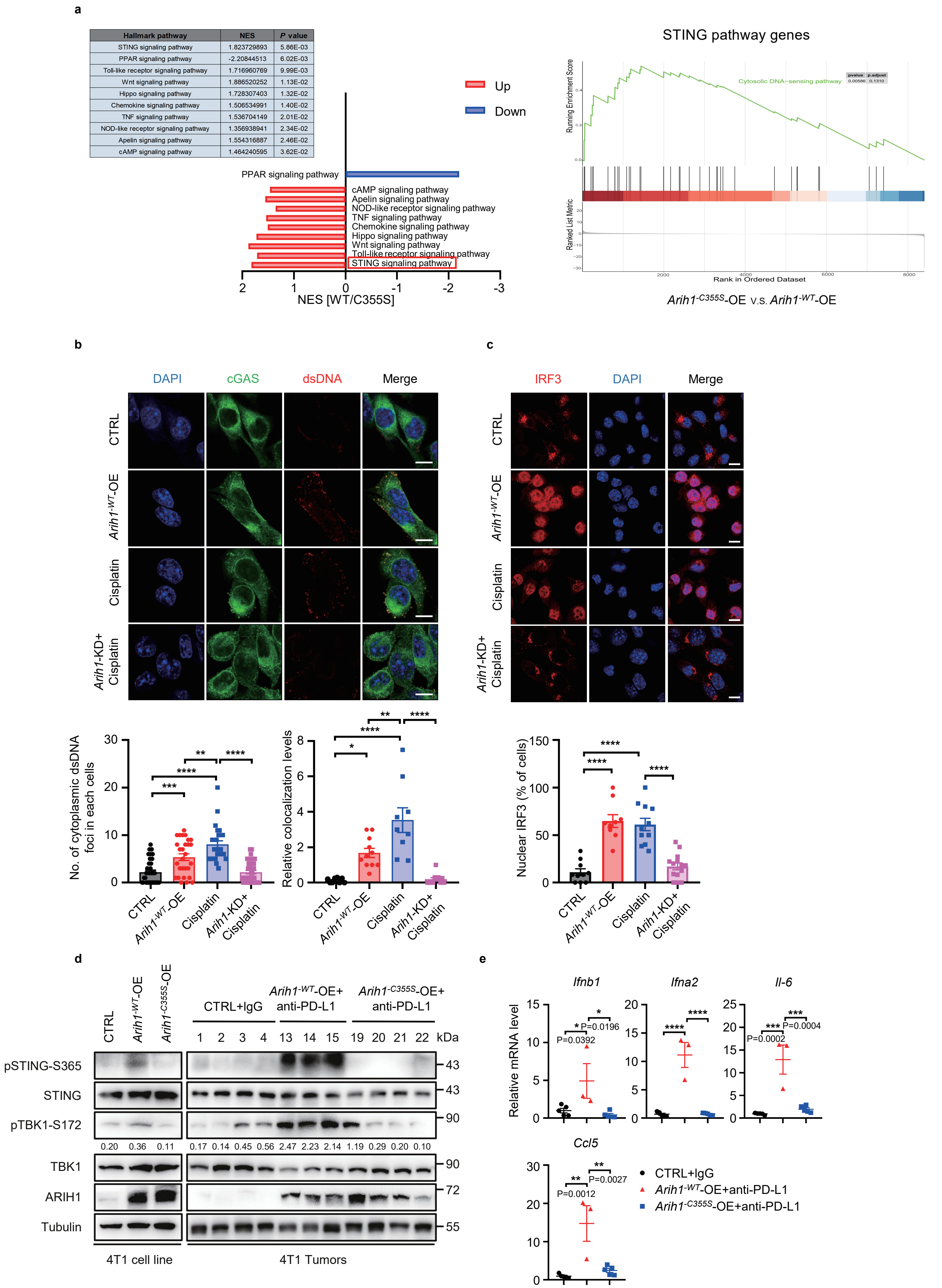
a
Hallmark pathway | NES | P value
STING signaling pathway | 1.823729893 | 5.86E-03
PPAR signaling pathway | -2.20844513 | 6.02E-03
Toll-like receptor signaling pathway | 1.716960769 | 9.99E-03
Wnt signaling pathway | 1.886520252 | 1.13E-02
Hippo signaling pathway | 1.728307403 | 1.32E-02
Chemokine signaling pathway | 1.506534991 | 1.40E-02
TNF signaling pathway | 1.536704149 | 2.01E-02
NOD-like receptor signaling pathway | 1.356938941 | 2.34E-02
Apelin signaling pathway | 1.554316887 | 2.46E-02
cAMP signaling pathway | 1.464240595 | 3.62E-02
Up
Down
PPAR signaling pathway
cAMP signaling pathway
Apelin signaling pathway
NOD-like receptor signaling pathway
TNF signaling pathway
Chemokine signaling pathway
Hippo signaling pathway
Wnt signaling pathway
Toll-like receptor signaling pathway
STING signaling pathway
NES [WT/C355S]
STING pathway genes
Running Enrichment Score
Cytosolic DNA−sensing pathway
pvalue p.adjust
0.00586 0.1310
Ranked List Metric
Rank in Ordered Dataset
Arih1-C355S-OE v.s. Arih1-WT-OE
b
DAPI
cGAS
dsDNA
Merge
CTRL
Arih1-WT-OE
Cisplatin
Arih1-KD+ Cisplatin
c
IRF3
DAPI
Merge
No. of cytoplasmic dsDNA foci in each cells
Relative colocalization levels
Nuclear IRF3 (% of cells)
d
CTRL
Arih1-WT-OE
Arih1-C355S-OE
CTRL+IgG
Arih1-WT-OE+ anti-PD-L1
Arih1-C355S-OE+ anti-PD-L1
1 2 3 4 13 14 15 19 20 21 22 kDa
pSTING-S365
STING
pTBK1-S172
TBK1
ARIH1
Tubulin
0.20 0.36 0.11 0.17 0.14 0.45 0.56 2.47 2.23 2.14 1.19 0.29 0.20 0.10
43
43
90
90
72
55
4T1 cell line
4T1 Tumors
e
Ifnb1
Ifna2
Il-6
Ccl5
Relative mRNA level
P=0.0392
P=0.0196
P=0.0002
P=0.0004
P=0.0012
P=0.0027
CTRL+IgG
Arih1-WT-OE+anti-PD-L1
Arih1-C355S-OE+anti-PD-L1

Supplementary Figure 9: The E3 ligase activity of ARIH1 is required for STING pathway activation.

a. GSEA analysis of the enrichment of top pathways (left) for genes between *Arih1*$^{-WT}$-OE and *Arih1*$^{-C355S}$-OE 4T1 cells (n=2/group). Representative upregulated GSEA plot with core-enriched genes of the STING pathway is shown (right). NES, normalized enrichment score. *p* values were calculated using Permutation tests. **b.** Immunofluorescence analysis of dsDNAs and cGAS in CTRL, *Arih1*$^{-WT}$-OE and *Arih1*-KD 4T1 cells with indicated treatments. The nuclei were stained with DAPI. Representative confocal images and quantitative data are shown. Scale bar, 10μm. Each dot in the graph represents the number of dsDNA foci counted per cell (left) and the average number of colocalized spots counted for each sample (right), and the total number of counted cells in each group is as follows: CTRL (n=36 cells), *Arih1*$^{-WT}$-OE (n=28 cells), Cisplatin (n=26 cells), and *Arih1*-KD+Cisplatin (n=42 cells). Data represent means ± SEM. Left **P < 0.01 (P = 0.0058), ***P < 0.001 (P = 0.0003), ****P < 0.0001. Right *P < 0.05 (P = 0.0229), **P < 0.01 (P = 0.0037), ****P < 0.0001. **c.** Immunofluorescent staining of IRF3 in CTRL, *Arih1*$^{-WT}$-OE and *Arih1*-KD 4T1 cells with indicated treatments and their quantifications. The nuclei were stained with DAPI. Scale bar, 10μm. Each dot in the graph represents the percentage of counted nucleus IRF3 cells in each sample, and the total number of counted cells in each group is as follows: CTRL (n=124 cells), *Arih1*$^{-WT}$-OE (n= 75 cells), Cisplatin (n= 86 cells), and *Arih1*-KD+Cisplatin (n=137 cells). Data represent means ± SEM, ****P < 0.0001. **d.** Immunoblot analysis of total and phospho STING (S365), total and phospho TBK1 (S172) in tumors of mice as in Figure 2**h**. The numbers under the blots represent the gray scale quantification (pTBK1-S172/TBK1). **e.** qRT-PCR measurement of tumor ISGs expression of the mice as in Figure 2**h**. n=5, 3, 5 mice/group. Data represent means ± SEM, *P < 0.05, **P < 0.01, ***P < 0.001, ****P < 0.0001. For **b-c** and **e** data, One-way ANOVA test. Data shown in **d** is representative of three independent experiments. Source data are provided as a Source Data file.

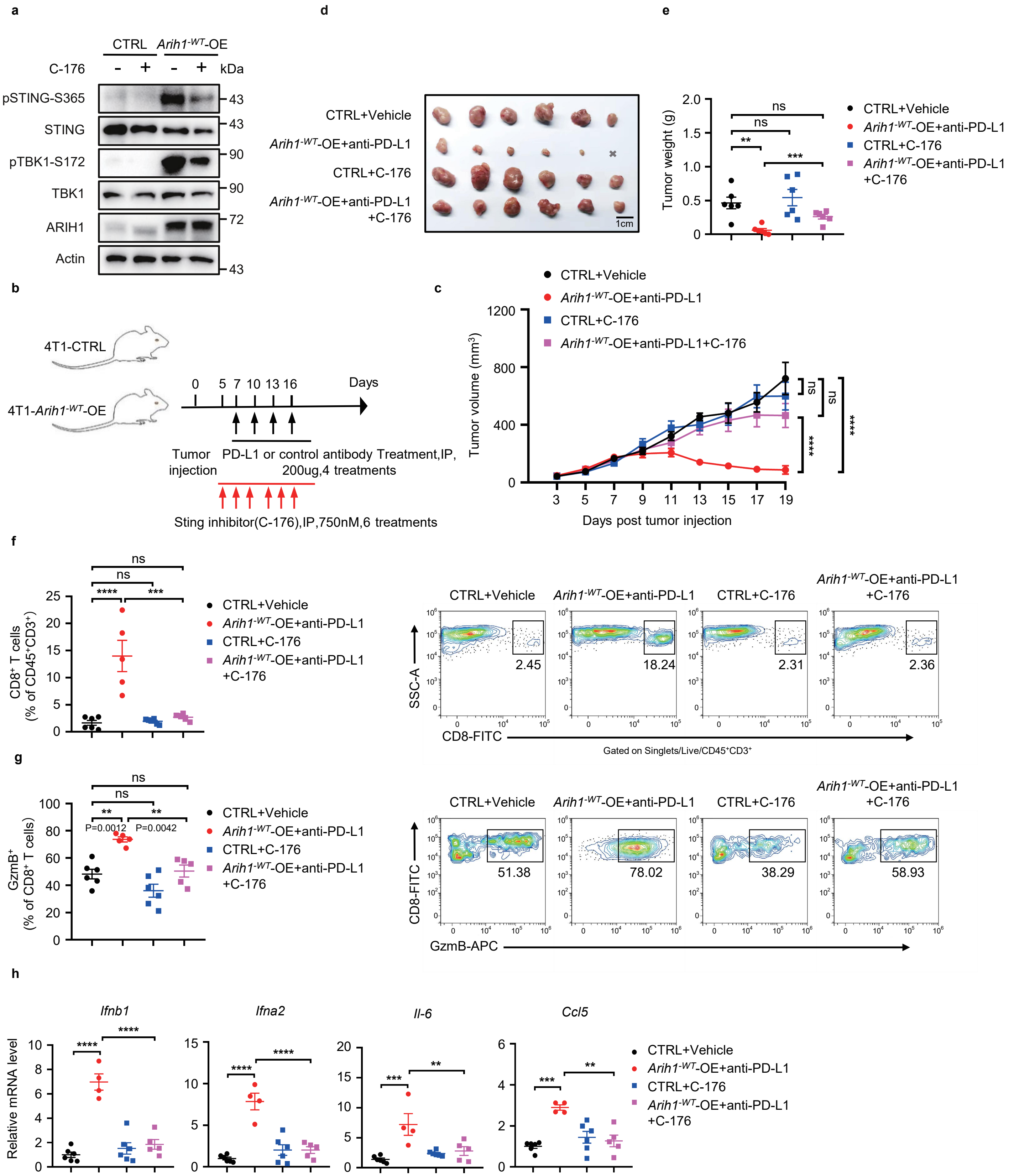

a
CTRL
Arih1-WT-OE
C-176
kDa
pSTING-S365
STING
pTBK1-S172
TBK1
ARIH1
Actin
43
43
90
90
72
43
b
4T1-CTRL
4T1-Arih1-WT-OE
0 5 7 10 13 16
Days
Tumor injection
PD-L1 or control antibody Treatment,IP, 200ug,4 treatments
Sting inhibitor(C-176),IP,750nM,6 treatments
c
CTRL+Vehicle
Arih1-WT-OE+anti-PD-L1
CTRL+C-176
Arih1-WT-OE+anti-PD-L1+C-176
Tumor volume (mm3)
Days post tumor injection
d
CTRL+Vehicle
Arih1-WT-OE+anti-PD-L1
CTRL+C-176
Arih1-WT-OE+anti-PD-L1 +C-176
1cm
e
Tumor weight (g)
f
CD8+ T cells (% of CD45+CD3+)
SSC-A
CD8-FITC
Gated on Singlets/Live/CD45+CD3+
2.45
18.24
2.31
2.36
g
GzmB+ (% of CD8+ T cells)
P=0.0012
P=0.0042
CD8-FITC
GzmB-APC
51.38
78.02
38.29
58.93
h
Ifnb1
Ifna2
Il-6
Ccl5
Relative mRNA level

Supplementary Figure 10: C-176, a STING inhibitor, reverses the anti-tumor effect of ARIH1-enhanced PD-L1 blockade therapy.

a. Immunoblots analysis of the STING pathway including total and phospho STING (S365) and total and phospho TBK1 (S172) in 4T1 cells with indicated treatments. **b.** A schematic model that illustrates the treatment plan for the female BALB/c mice (6-8 week old) bearing CTRL and *Arih1^{-WT}*-OE 4T1 tumors (n=6 per group) with indicated treatments.This image was created by the first author. **c.** Tumor growth curves of CTRL and *Arih1^{-WT}*-OE 4T1 cells in female BALB/c mice (n=6 per group, 6-8 week old) with indicated treatments. Data represent means ± SEM, ****P < 0.0001, ns, not significant. **d-e.** Representative tumor image (**d**) of CTRL and *Arih1^{-WT}*-OE tumors in BALB/c mice with indicated treatments and final tumor weights (**e**). n=6 mice/group. Data represent means ± SEM, **P < 0.01 (P = 0.0012), ***P < 0.001 (P = 0.0010), ns, not significant. **f-g.** Representative figures and summary of frequency of tumor infiltrating $CD8^+$ T cells (**f**) and $GzmB^+CD8^+$ T cells (**g**) of mice as in (**c**). n=6, 5, 6, 5 mice/group. Data represent means ± SEM, **f** ***P < 0.001 (P = 0.0001), ****P < 0.0001, ns, not significant. **h.** qRT-PCR measurement of tumor ISGs expression of the mice as in (**c**). n=6, 4, 6, 5 mice/group. Data represent means ± SEM. *Ifnb1* ****P < 0.0001. *Ifna2* ****P < 0.0001. *Il6* **P < 0.01 (P = 0.0077), ***P < 0.001 (P = 0.0004). *Ccl5* **P < 0.01 (P = 0.0011), ***P < 0.001 (P = 0.0001). For **c** data, Two-way ANOVA test. For **f-h** data, One-way ANOVA test. For **e** data, Two-tailed t-test. Data shown in **g** is representative of two independent experiments. Data shown in **a** is representative of three independent experiments. Source data are provided as a Source Data file.

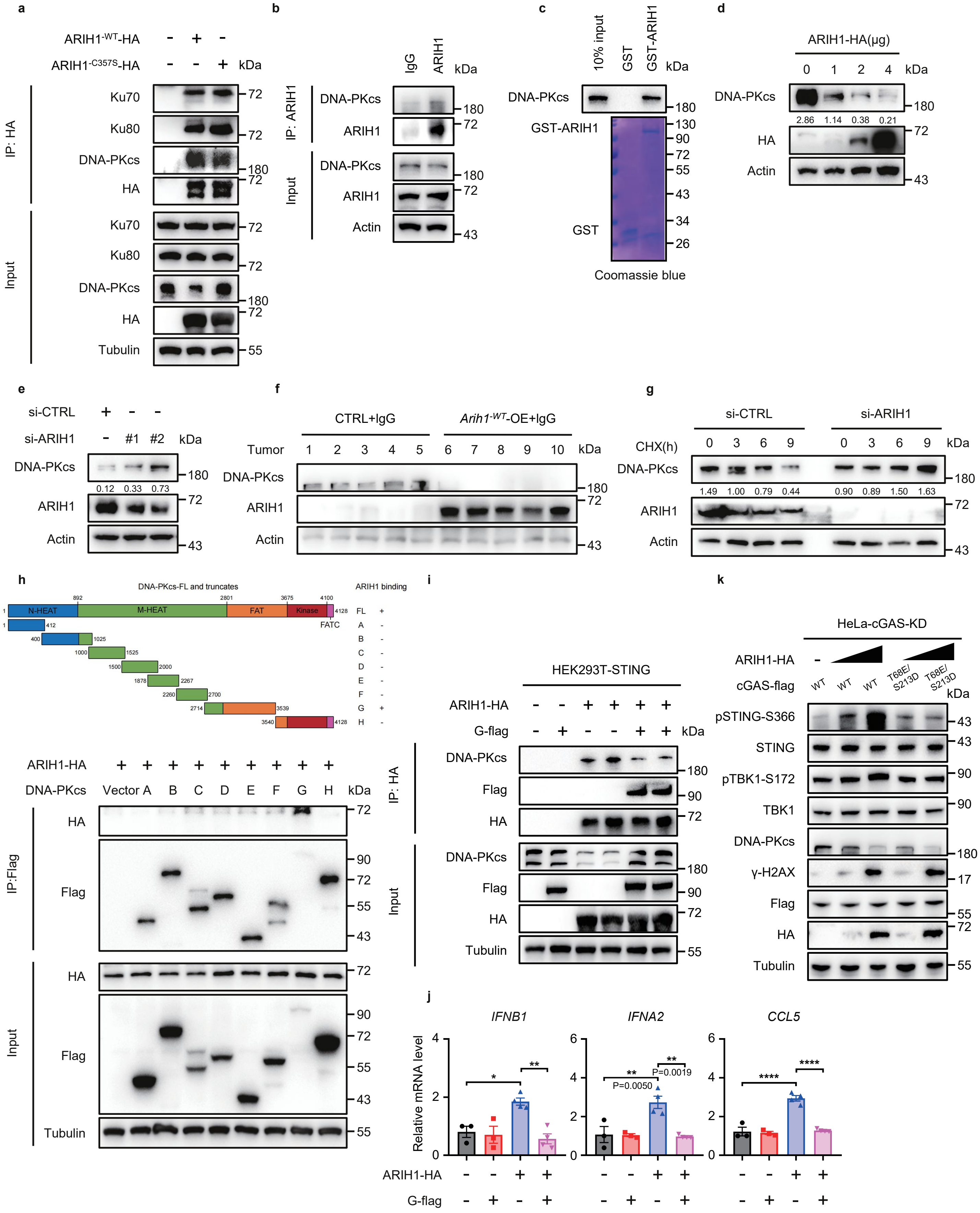

a
ARIH1-WT-HA - + -
ARIH1-C357S-HA - - +
kDa
IP: HA
Ku70
Ku80
DNA-PKcs
HA
Input
Ku70
Ku80
DNA-PKcs
HA
Tubulin
72
180
55
b
IgG
ARIH1
kDa
IP: ARIH1
DNA-PKcs
ARIH1
Input
DNA-PKcs
ARIH1
Actin
180
72
43
c
10% input
GST
GST-ARIH1
kDa
DNA-PKcs
GST-ARIH1
GST
Coomassie blue
180
130
90
72
55
43
34
26
d
ARIH1-HA(μg)
0 1 2 4
kDa
DNA-PKcs
2.86 1.14 0.38 0.21
HA
Actin
180
72
43
e
si-CTRL + - -
si-ARIH1 - #1 #2
kDa
DNA-PKcs
0.12 0.33 0.73
ARIH1
Actin
180
72
43
f
CTRL+IgG
Arih1-WT-OE+IgG
Tumor 1 2 3 4 5 6 7 8 9 10
kDa
DNA-PKcs
ARIH1
Actin
180
72
43
g
si-CTRL
si-ARIH1
CHX(h) 0 3 6 9 0 3 6 9
kDa
DNA-PKcs
1.49 1.00 0.79 0.44 0.90 0.89 1.50 1.63
ARIH1
Actin
180
72
43
h
DNA-PKcs-FL and truncates
ARIH1 binding
N-HEAT
M-HEAT
FAT
Kinase
FATC
1 892 2801 3675 4100 4128
FL +
A -
B -
C -
D -
E -
F -
G +
H -
1 412
400 1025
1000 1525
1500 2000
1878 2267
2260 2700
2714 3539
3540 4128
ARIH1-HA + + + + + + + + +
DNA-PKcs Vector A B C D E F G H
kDa
IP:Flag
HA
Flag
Input
HA
Flag
Tubulin
72
90
55
43
i
HEK293T-STING
ARIH1-HA - - + + + +
G-flag - + - - + +
kDa
IP: HA
DNA-PKcs
Flag
HA
Input
DNA-PKcs
Flag
HA
Tubulin
180
90
72
55
j
IFNB1
IFNA2
CCL5
Relative mRNA level
*
**
P=0.0050
P=0.0019
****
ARIH1-HA - - + +
G-flag - + - +
k
HeLa-cGAS-KD
ARIH1-HA
cGAS-flag WT WT WT T68E/S213D T68E/S213D
kDa
pSTING-S366
STING
pTBK1-S172
TBK1
DNA-PKcs
γ-H2AX
Flag
HA
Tubulin
43
90
180
17
55
72

Supplementary Figure 11: ARIH1-mediated degradation of DNA-PKcs promotes STING pathway activation.

a. Co-IP analysis for the interaction of ARIH1^{-WT}-HA or ARIH1^{-C357S}-HA (HA) with endogenous Ku80, Ku70 and DNA-PKcs in HEK293T cells. **b.** Co-IP of DNA-PKcs with ARIH1 in HEK293T cells. Endogenous ARIH1 was immunoprecipitated using anti-ARIH1, and the immunoprecipitates were analyzd with anti-DNA-PKcs. IgG, immunoglobulin G. **c.** Immunoblots (IB) analysis of glutathione S-transferase (GST) pull-down precipitates from HEK293T cell lysates with endogenous DNA-PKcs and bacterially purified recombinant GST or GST-ARIH1 protein. **d-e.** IB analysis of DNA-PKcs levels in HeLa cells. The cells were incubated with small interfering RNAs (siRNAs) against ARIH1 (**e**), or transfected with ARIH1-HA (**d**). **f.** IB analysis of DNA-PKcs levels in the 4T1 tumor lysate in Figure 2**a** was performed. **g.** IB analysis of DNA-PKcs levels in 40μg/mL cycloheximide (CHX)-treated HeLa cells transfected with indicated constructs. **h.** The schematic illustration of DNA-PKcs protein sequence with truncated mutants. Co-IP of ARIH1 (HA) with different DNA-PKcs truncation proteins (Flag) as indicated. **i.** Co-IP of ARIH1 with DNA-PKcs or its truncated mutant (G-Flag) in HEK293T-STING cells. Exogenous ARIH1 was immunoprecipitated using anti-HA, and the immunoprecipitates were analyzd with anti-DNA-PKcs and anti-Flag. **j.** The HEK293T-STING cells were co-transfected with indicated constructs to detect ISGs expression by qRT-PCR. n=3, 3, 4, 4 per group. Data represent means ± SEM. *IFNB1* *P < 0.05 (P = 0.0153), **P < 0.01 (P = 0.0023). *IFNA2* **P < 0.01. *CCL5* ****P < 0.0001. **k.** cGAS-KD HeLa cells were transfected with cGAS WT or the phosphorylation-mimic mutants and ARIH1-HA. WCLs were analyzed by immunoblotting. For **a-f**, **h-i**, and **k**, two independent expriments are conducted. For **g** and **j**, at least three independent expriments are conducted. In **d-e** and **g**, the numbers under the blots represent the gray scale quantification (DNA-PKcs/Actin). For **j** data, One-way ANOVA test. Source data are provided as a Source Data file.

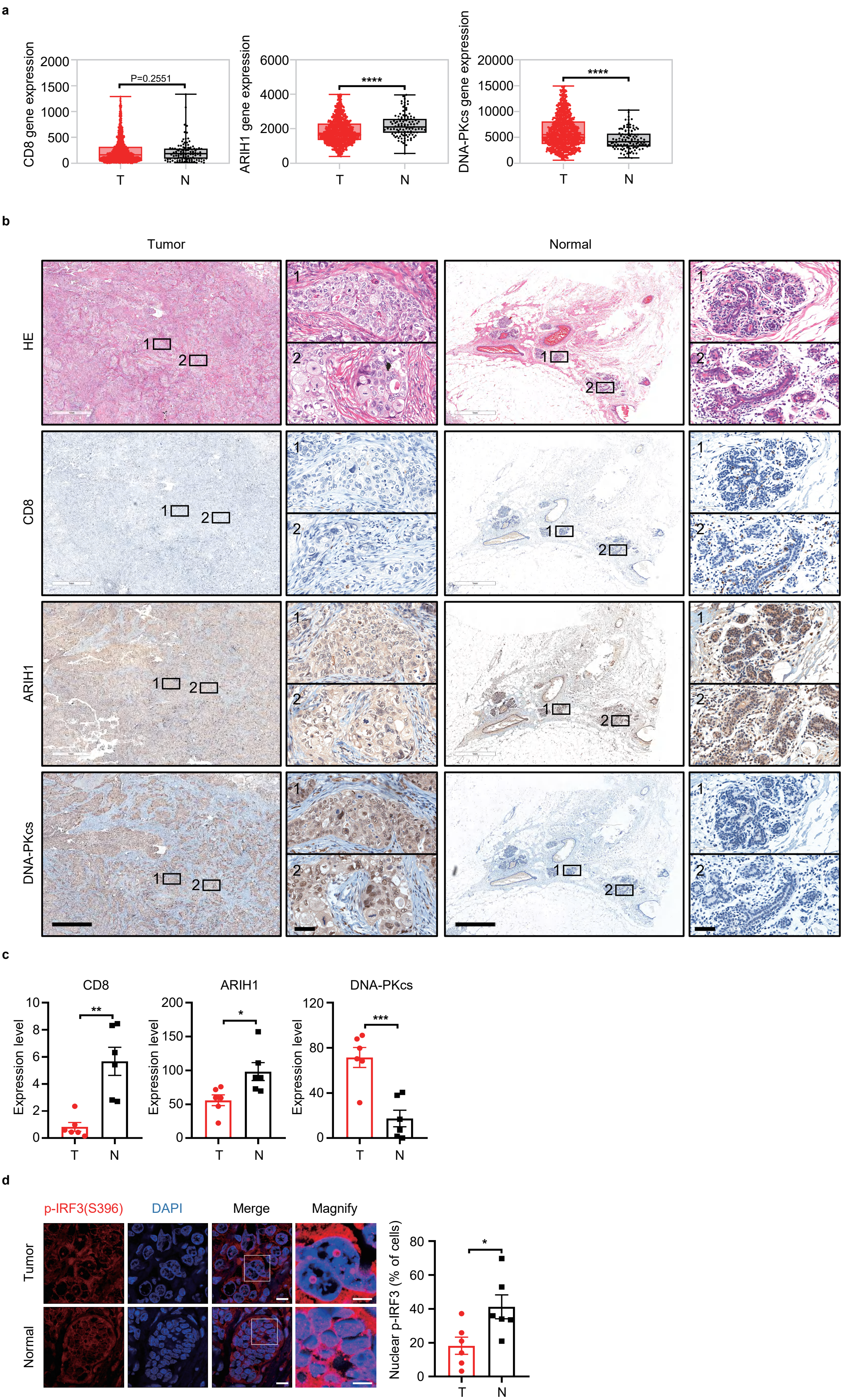
a
CD8 gene expression
P=0.2551
T
N
ARIH1 gene expression
****
DNA-PKcs gene expression
****
b
Tumor
Normal
HE
CD8
ARIH1
DNA-PKcs
c
CD8
**
ARIH1
*
DNA-PKcs
***
Expression level
d
p-IRF3(S396)
DAPI
Merge
Magnify
Tumor
Normal
Nuclear p-IRF3 (% of cells)
*

Supplementary Figure 12: Reduced ARIH1 expression shows DNA-PKcs accumulation and inhibition of STING pathway in human breast cancer biopsies.

a. Analysis of RNA-sep data of CD8, ARIH1 and DNA-PKcs in human breast invasive carcinoma and normal tissues from the TNM plot platform (https://tnmplot.com/analysis/). Box plots indicate median (middle line), 25th, 75th percentile (box) and minima and maxima (whiskers). CD8 (n=1056,113/group), ARIH1 (n=1039,107/group), DNA-PKcs (n=989,113/group). Data represent means ± SEM, ****P < 0.0001. **b.** Representative images of CD8, ARIH1 and DNA-PKcs IHC staining from human TNBC and paracancerous normal tissue specimens. The areas indicated by boxes are magnified on the right. Scale bars, 1mm (main images) and 60μm (magnified images). **c.** Quantification of IHC analysis (n=6 per group) for CD8, ARIH1 and DNA-PKcs. Data represent means ± SEM, *P < 0.05 (P = 0.0210), **P < 0.01 (P = 0.0012), ***P < 0.001 (P = 0.0008). **d.** Immunostaining of pIRF3-S396 in TNBC tumors and normal sample specimens (n=6 per group) and their quantifications. The nuclei were stained with DAPI. T (n=537 cells), N (n=439 cells). Scale bar, 10μm; insets: Scale bar, 5μm. Data represent means ± SEM, *P < 0.05 (P = 0.0248). For **a, c, d** data, Two-tailed t-test. Source data are provided as a Source Data file.

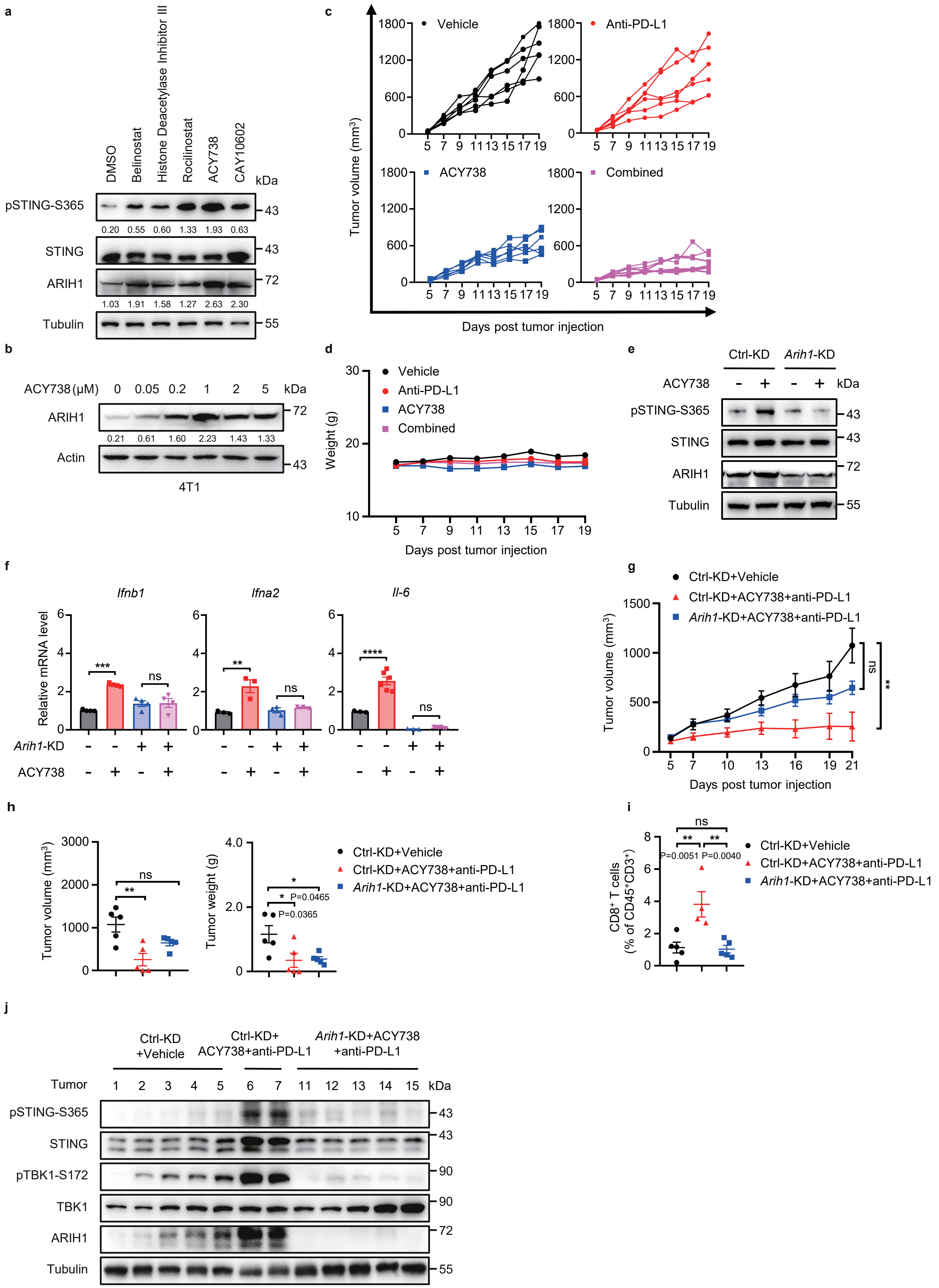

a
DMSO
Belinostat
Histone Deacetylase Inhibitor III
Rocilinostat
ACY738
CAY10602
kDa
pSTING-S365
0.20 0.55 0.60 1.33 1.93 0.63
STING
ARIH1
1.03 1.91 1.58 1.27 2.63 2.30
Tubulin
b
ACY738 (μM) 0 0.05 0.2 1 2 5
ARIH1
0.21 0.61 1.60 2.23 1.43 1.33
Actin
4T1
c
Vehicle
Anti-PD-L1
ACY738
Combined
Tumor volume (mm³)
Days post tumor injection
d
Weight (g)
Days post tumor injection
e
Ctrl-KD
Arih1-KD
ACY738
pSTING-S365
STING
ARIH1
Tubulin
f
Ifnb1
Ifna2
Il-6
Relative mRNA level
Arih1-KD
ACY738
g
Ctrl-KD+Vehicle
Ctrl-KD+ACY738+anti-PD-L1
Arih1-KD+ACY738+anti-PD-L1
Tumor volume (mm³)
Days post tumor injection
h
Tumor volume (mm³)
Tumor weight (g)
P=0.0465
P=0.0365
i
CD8⁺ T cells (% of CD45⁺CD3⁺)
P=0.0051
P=0.0040
j
Ctrl-KD +Vehicle
Ctrl-KD+ ACY738+anti-PD-L1
Arih1-KD+ACY738 +anti-PD-L1
Tumor 1 2 3 4 5 6 7 11 12 13 14 15
pSTING-S365
STING
pTBK1-S172
TBK1
ARIH1
Tubulin

Supplementary Figure 13: Knockdown of ARIH1 reverses the anti-tumor effect of PD-L1 blockade with ACY738.

a. Immunoblots (IB) of pSTING-S365 and ARIH1 in 4T1 cells treated with indicated drugs at 5μM for 24 hours. The numbers under the blots represent the gray scale quantification (pSTING-S365 /STING, ARIH1/Tubulin). **b.** IB analysis of ARIH1 levels in 4T1 cells after treatment with ACY738 24 hours for the indicated concentration. The numbers under the blots represent the gray scale quantification (ARIH1/Actin). **c.** Tumor growth curves for each tumor bearing BALB/c mouse (n=6 per group) in Figure 6**c**. **d.** Representative body weights of tumor bearing mice in Figure 6**c**. n=6 mice/group. **e.** IB of pSTING-S365 and ARIH1 in Ctrl-KD and *Arih1*-KD 4T1 cells treated with ACY738 1μM for 24 hours. **f.** qRT-PCR measurement of ISGs expression in Ctrl-KD compared to *Arih1*-KD 4T1 cells with indicated treatments. *Ifnb1 (*n=4/group), *Ifna2 (*n=3/group), *Il6 (*n=3, 6, 3, 6/group). Data represent means ± SEM, **P < 0.01 (P = 0.0031), ***P < 0.001 (P = 0.0001), ****P < 0.0001, ns, not significant. **g-h.** Tumor growth curves (**g**), final tumor volume (left) and tumor weights (right) (**h**) in Ctrl-KD and *Arih1*-KD 4T1 cells in female BALB/c mice (n=5 per group, 6-8 week old) with indicated treatments. Data represent means ± SEM. **g** **P < 0.01 (P = 0.0028), ns, not significant. **h** *P < 0.05, **P < 0.01 (P = 0.0031), ns, not significant. **i.** Quantification of tumor-infiltrating $CD8^+$ T cells in Ctrl-KD and *Arih1*-KD 4T1 tumors of the mice as in (**g**). n=5, 4, 5 mice/group. Data represent means ± SEM, **P < 0.01, ns, not significant. **j.** IB analysis of pSTING-S365, pTBK1-S172, and ARIH1 of the indicated tumor lysates in the experiment described in (**g**). For **g** data, Two-way ANOVA test. For **f** and **h-i** data, One-way ANOVA test. Data shown in **a-b**, **e**, and **j** are representative of three independent experiments. Source data are provided as a Source Data file.

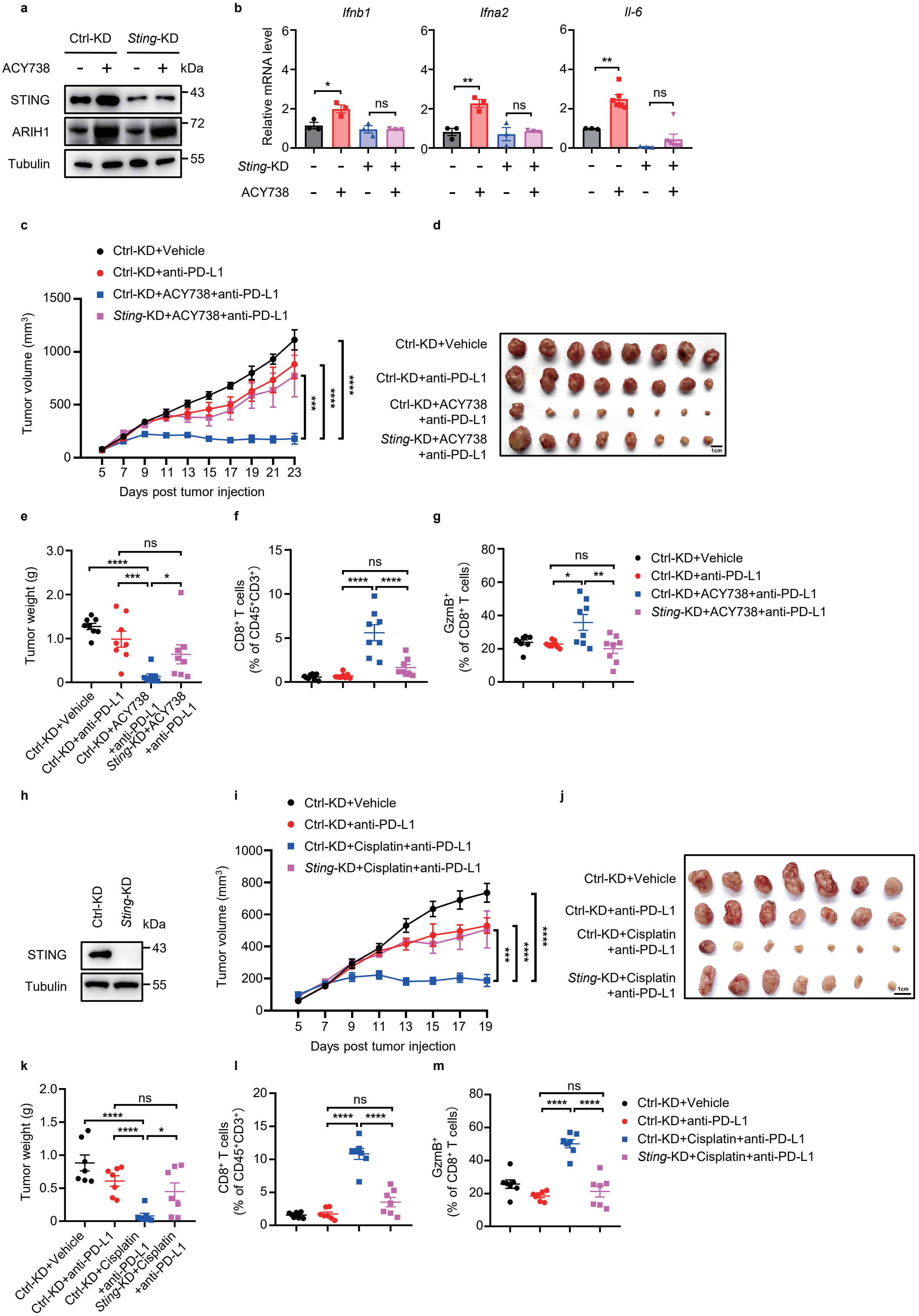
a
Ctrl-KD
Sting-KD
ACY738
kDa
STING
ARIH1
Tubulin
43
72
55
b
Ifnb1
Ifna2
Il-6
Relative mRNA level
Sting-KD
ACY738
ns
c
Ctrl-KD+Vehicle
Ctrl-KD+anti-PD-L1
Ctrl-KD+ACY738+anti-PD-L1
Sting-KD+ACY738+anti-PD-L1
Tumor volume (mm³)
Days post tumor injection
d
Ctrl-KD+Vehicle
Ctrl-KD+anti-PD-L1
Ctrl-KD+ACY738 +anti-PD-L1
Sting-KD+ACY738 +anti-PD-L1
1cm
e
Tumor weight (g)
f
CD8⁺ T cells (% of CD45⁺CD3⁺)
g
GzmB⁺ (% of CD8⁺ T cells)
h
Ctrl-KD
Sting-KD
kDa
STING
Tubulin
43
55
i
Ctrl-KD+Vehicle
Ctrl-KD+anti-PD-L1
Ctrl-KD+Cisplatin+anti-PD-L1
Sting-KD+Cisplatin+anti-PD-L1
Tumor volume (mm³)
Days post tumor injection
j
Ctrl-KD+Cisplatin +anti-PD-L1
Sting-KD+Cisplatin +anti-PD-L1
1cm
k
Tumor weight (g)
l
CD8⁺ T cells (% of CD45⁺CD3⁺)
m
GzmB⁺ (% of CD8⁺ T cells)

Supplementary Figure 14: Knockdown of STING reverses the anti-tumor effect of immune checkpoint inhibition and therapeutic agents.

a. Immunoblot (IB) analysis of STING and ARIH1 in Ctrl-KD and *Sting*-KD 4T1 cells treated with ACY738 1μM for 24 hours. **b.** qRT-PCR measurement of ISGs expression in Ctrl-KD compared to *Sting*-KD 4T1 cells with indicated treatments. *Ifnb1 (*n=3/group), *Ifna2 (*n=3/group), *Il6 (*n=3, 6, 3, 6/group). Data represent means ± SEM, *Ifnb1* *P < 0.05 (P = 0.0243), *Ifna2* **P < 0.01 (P = 0.0066), *Il6* **P < 0.01 (P = 0.0032), ns, not significant. **c-e.** Tumor growth curves (**c**), final tumor image (**d**) and tumor weights (**e**) in Ctrl-KD and *Sting*-KD 4T1 cells in female BALB/c mice (n=8 per group, 6-8 week old) with indicated treatments. Data represent means ± SEM. **c** ***P < 0.001 (P = 0.0009), ****P < 0.0001. **e** *P < 0.05 (P = 0.0443), ***P < 0.001 (P = 0.0006), ****P < 0.0001, ns, not significant. **f-g.** Quantification of tumor-infiltrating $CD8^+$ T cells (**f**) and $GzmB^+CD8^+$ T cells (**g**) in Ctrl-KD and *Sting*-KD 4T1 tumors of the mice as in (**c**). n=8 mice/group. Data represent means ± SEM, *P < 0.05 (P = 0.0168), **P < 0.01 (P = 0.0031), ****P < 0.0001, ns, not significant. **h.** IB analysis of STING levels in Ctrl-KD and *Sting*-KD 4T1 cells. **i-k.** Tumor growth curves (**i**), final tumor image (**j**) and tumor weights (**k**) of Ctrl-KD and *Sting*-KD 4T1 cells in female BALB/c mice (n=7 per group, 6-8 week old) with indicated treatments. Data represent means ± SEM. **i** ***P < 0.001 (P = 0.0005), ****P < 0.0001. **k** *P < 0.05 (P = 0.0211), ****P < 0.0001, ns, not significant. **l-m.** Quantification of tumor-infiltrating $CD8^+$ T cells (**l**) and $GzmB^+CD8^+$ T cells (**m**) in Ctrl-KD and *Sting*-KD 4T1 tumors of the mice as in (**i**). n=7 mice/group. Data represent means ± SEM, ****P < 0.0001, ns, not significant. For **c** and **i** data, Two-way ANOVA test. For **b**, **f-g**, and **l-m** data, One-way ANOVA test. For **e** and **k** data, Two-tailed t-test. Data shown in **a** is representative of two independent experiments. Data shown in **h** is representative of three independent experiments. Source data are provided as a Source Data file.

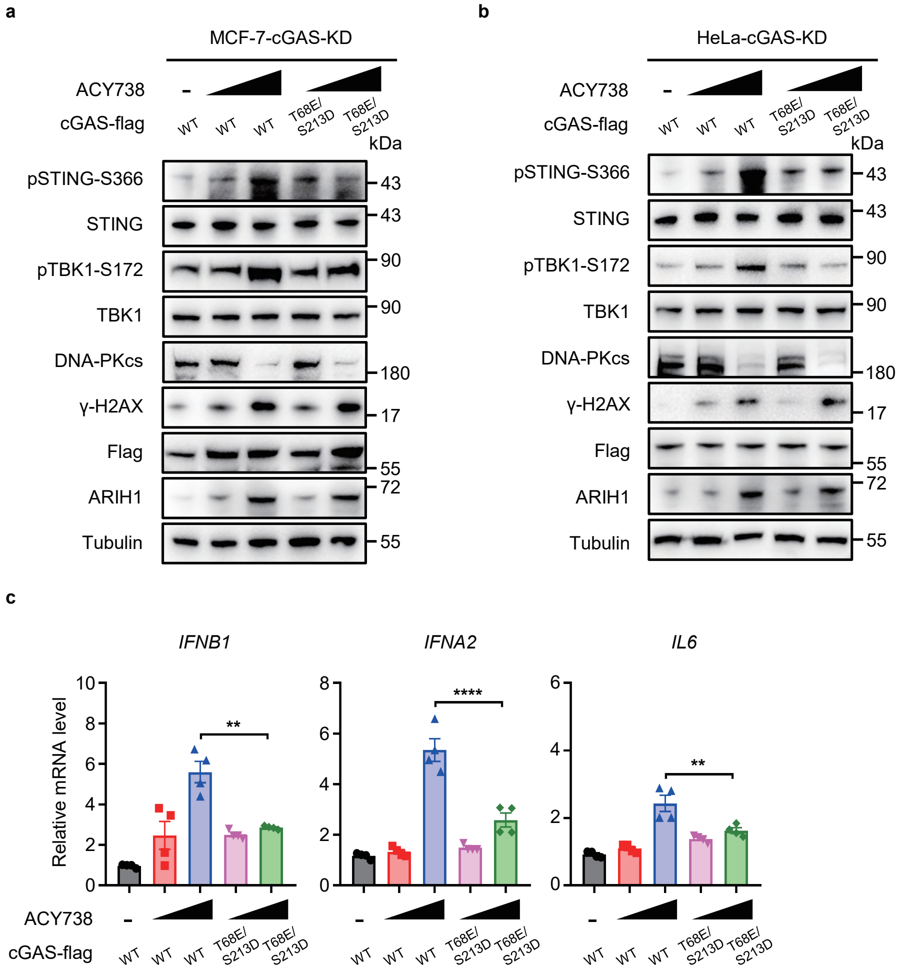

Supplementary Figure 15: The phosphorylation-mimetic mutant T68E/S213D of cGAS inhibits the activation of STING pathway mediated by ACY738.

a-b. cGAS-KD MCF-7 or HeLa cells were transfected with cGAS WT or the phosphorylation-mimic mutants and then treated with ACY738 for 24 hours. WCLs were analyzed by immunoblotting. **c.** qRT-PCR measurement of ISGs expression in cGAS-KD HeLa cells transfecting with cGAS WT or the phosphorylation-mimic mutants and then treating with ACY738 for 24 hours. n=4/group. Data represent means ± SEM. *IFNB1* **P < 0.01 (P = 0.0014), *IFNA2* ****P < 0.0001, *IL6* **P < 0.01 (P = 0.0022). For **a-b**, two independent expriments are conducted. For **c** data, One-way ANOVA test. Source data are provided as a Source Data file.

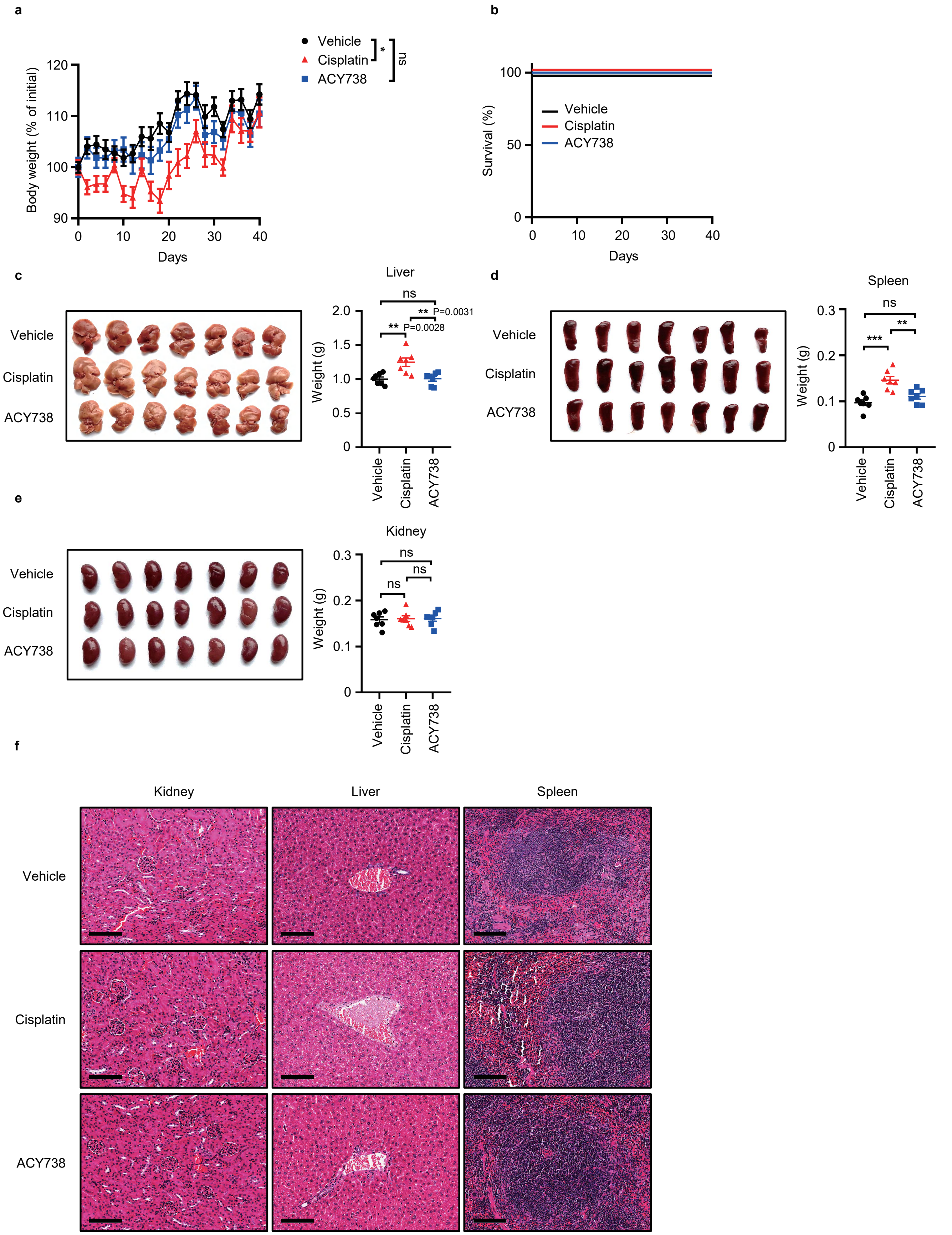
a
Body weight (% of initial)
Days
Vehicle
Cisplatin
ACY738
*
ns
b
Survival (%)
Days
Vehicle
Cisplatin
ACY738
c
Liver
Vehicle
Cisplatin
ACY738
Weight (g)
ns
** P=0.0031
** P=0.0028
d
Spleen
Vehicle
Cisplatin
ACY738
Weight (g)
ns
***
**
e
Kidney
Vehicle
Cisplatin
ACY738
Weight (g)
ns
ns
ns
f
Kidney
Liver
Spleen
Vehicle
Cisplatin
ACY738

Supplementary Figure 16: ACY738 is a potent anti-tumor agent without detectable toxicity to mice.

a-b. The female BALB/c mice (n=7 per group, 6-8 week old) were treated with saline (vehicle), cisplatin (5mg/kg, 1 out of 7 days), or ACY738 (5mg/kg, daily) for three weeks. Their body weights (**a**) and their survival rates (**b**) were monitored every day. Data represent means ± SEM. **a** *P < 0.05 (P = 0.0412), ns, not significant.**c-e.** On day 40, livers (**c**), spleens (**d**) and kidneys (**e**) were removed and organ weights were recorded. n=7/group. Data represent means ± SEM. **d** **P < 0.01 (P = 0.0037), ***P < 0.001 (P = 0.0002), ns, not significant. **f.** HE staining of kidneys, livers and spleens dissected from (**c**, **d**, **e**). Scale bar, 100μm. For **a** data, Two-way ANOVA test. For **c-e** data, One-way ANOVA test. Source data are provided as a Source Data file.

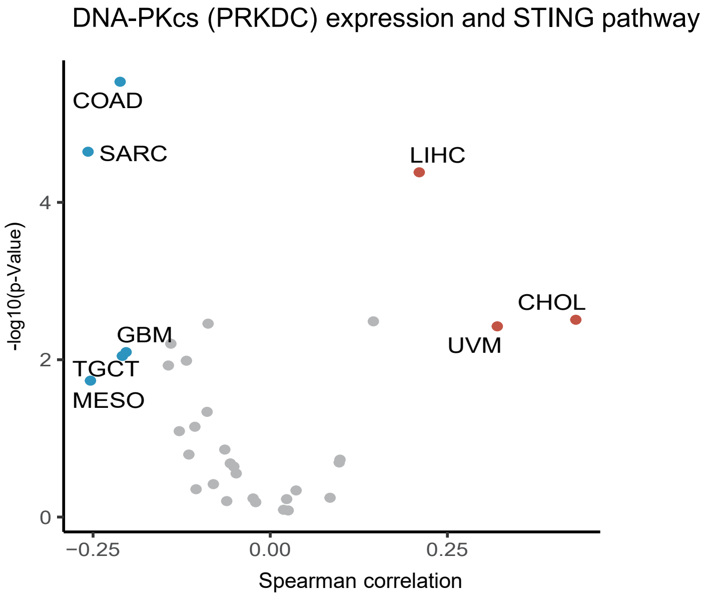

Supplementary Figure 17: Expression of DNA-PKcs (PRKDC) in human cancers.

Correlation of DNA-PKcs mRNA levels with the STING pathway from TCGA RNA-sep datasets across human cancer types. Plots show Spearman's correlation and estimated statistical significance for the indicated pathway. CHOL (Cholangio carcinoma, n=36 samples), COAD (Colon Adenocarcinoma, n=456 samples), GBM (Glioblastoma Multiforme, n=166 samples), LIHC (Liver Hepatocellular Carcinoma, n=371 samples), MESO (Mesothelioma, n=86 samples), SARC (Sarcoma, n=259 samples), TGCT (Testicular Germ Cell Tumors, n=150 samples), and UVM (Uveal Melanoma, n=80 samples).

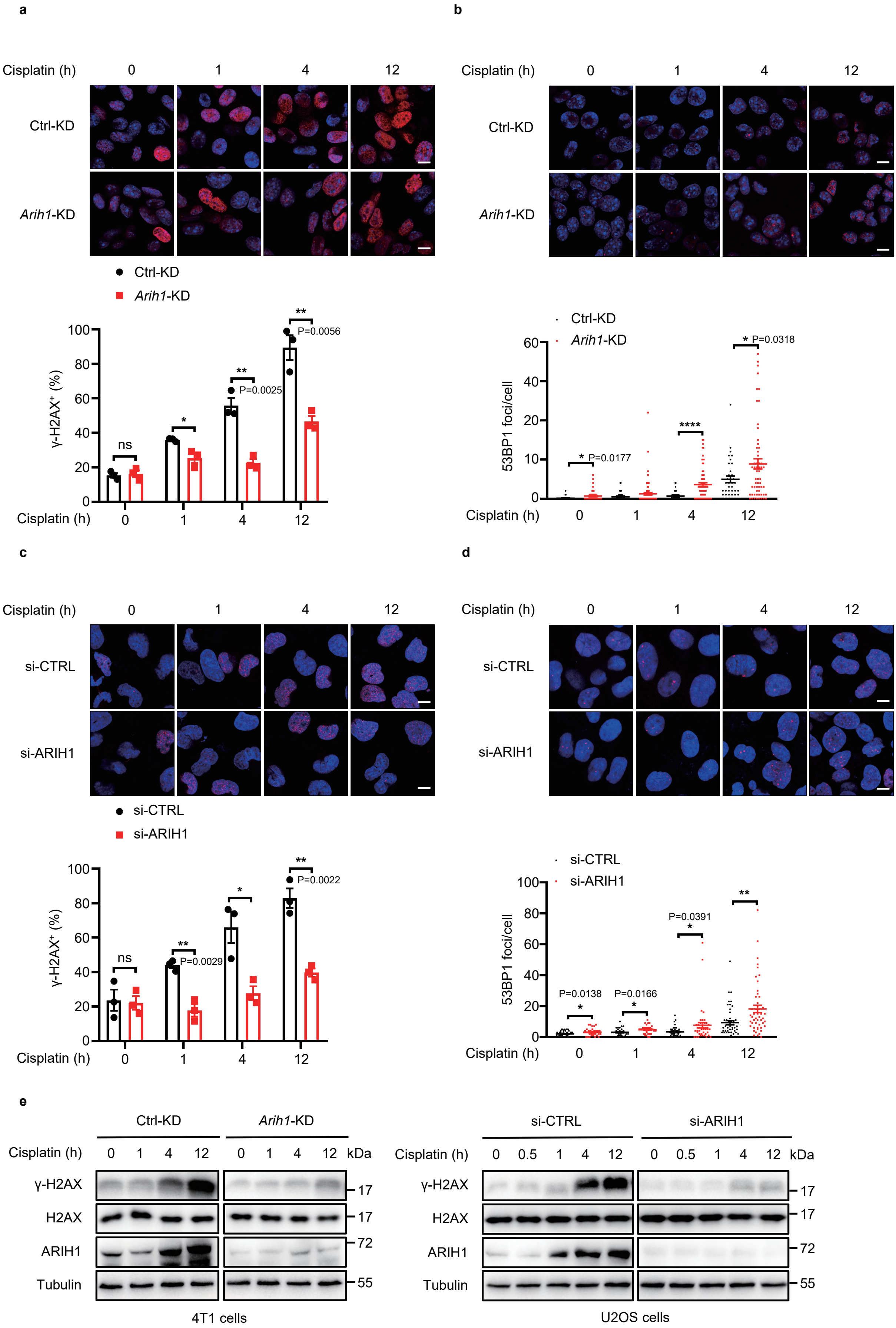
a
Cisplatin (h) 0 1 4 12
Ctrl-KD
Arih1-KD
Ctrl-KD
Arih1-KD
γ-H2AX⁺ (%)
ns
*
**
P=0.0025
**
P=0.0056
Cisplatin (h) 0 1 4 12
b
Cisplatin (h) 0 1 4 12
Ctrl-KD
Arih1-KD
Ctrl-KD
Arih1-KD
53BP1 foci/cell
* P=0.0177
****
* P=0.0318
Cisplatin (h) 0 1 4 12
c
Cisplatin (h) 0 1 4 12
si-CTRL
si-ARIH1
si-CTRL
si-ARIH1
γ-H2AX⁺ (%)
ns
**
P=0.0029
*
**
P=0.0022
Cisplatin (h) 0 1 4 12
d
Cisplatin (h) 0 1 4 12
si-CTRL
si-ARIH1
si-CTRL
si-ARIH1
53BP1 foci/cell
P=0.0138
P=0.0166
P=0.0391
**
Cisplatin (h) 0 1 4 12
e
Ctrl-KD
Arih1-KD
Cisplatin (h) 0 1 4 12 0 1 4 12 kDa
γ-H2AX
H2AX
ARIH1
Tubulin
17
17
72
55
4T1 cells
si-CTRL
si-ARIH1
Cisplatin (h) 0 0.5 1 4 12 0 0.5 1 4 12 kDa
γ-H2AX
H2AX
ARIH1
Tubulin
17
17
72
55
U2OS cells

Supplementary Figure 18: ARIH1 can affect cisplatin-induced DNA damage.

a-b. Immunofluorescence analysis of γ-H2AX (**a**) and 53BP1 (**b**) in Ctrl-KD and *Arih1*-KD 4T1 cells at the indicated times following cisplatin treatment (red, γ-H2AX/53BP1; blue, DAPI). Representative confocal images and quantitative data are shown. Scale bar, 10μm. Data represent means ± SEM. **a** *P < 0.05 (P = 0.0220), **P < 0.01, ns, not significant. **b** *P < 0.05, ****P < 0.0001. **c-d.** Immunofluorescence analysis of γ-H2AX (**c**) and 53BP1 (**d**) in U2OS cells transfected with ARIH1-siRNA (si-ARIH1) or scrambled siRNA (si-CTRL) at the indicated times following cisplatin treatment (red, γ-H2AX/53BP1; blue, DAPI). Representative confocal images and quantitative data are shown. Scale bar, 10μm. Data represent means ± SEM. **c** *P < 0.05 (P = 0.0188), **P < 0.01, ns, not significant. **d** *P < 0.05, **P < 0.01 (P = 0.0018). **e.** Immunoblots analysis of γ-H2AX and ARIH1 levels in 4T1 and U2OS cells after treatment with 10 μM cisplatin for indicated times. For **a-d** data, Two-tailed t-test. For **a** and **c** data, each dot in the graph represents the percentage of counted γ-H2AX$^+$ cells in each sample, and the total number of counted cells in each group is as follows: **a** (Ctrl-KD (n=616, 579, 607, 549 cells/group), *Arih1*-KD (n=515, 491, 467, 693 cells/group)). **c** (si-CTRL (n=276, 231, 311, 109 cells/group), si-ARIH1 (n=112, 164, 169, 133 cells/group)). For **b** and **d** data, each dot in the graph represents the number of 53BP1 foci counted per cell, and the total number of counted cells in each group is as follows: **b** (Ctrl-KD (n=31, 44, 48, 35 cells/group), *Arih1*-KD (n=56, 89, 72, 59 cells/group)). **d** (si-CTRL (n=47, 30, 35, 49 cells/group), si-ARIH1 (n=32, 34, 43, 49 cells/group)). Data shown in **e** is representative of three independent experiments. Source data are provided as a Source Data file.

siRNA primers (5' to 3') for human genes:	
ARIH1#1	CGAGAUAUUUCCCAAGAUUUU
ARIH1#2	CCAUGUUGUUAAAGUCCAAUA
cGAS#1	AGAGAAAUGUUGCAGGAAAGA
Control	UUCUCCGAACGUGUCACGUTT
shRNA primers (5' to 3') for mouse genes:	
Arih1#1	TACCTCTTGATCTAGTTGC
Sting#1	TTAAGAAGGCAGTTGACAG
Control	CCTAAGGTTAAGTCGCCCTCG

Supplementary Table 1. The siRNA and shRNA primers used in this study.

Q-PCR primers (5’ to 3’) for human genes:		
IFNB1	CAGGAGAGCAATTTGGAGGA	CTTTCGAAGCCTTTGCTCTG
IFNA2	CTTGTGCCTGGGAGGTTGTC	AAAAGGTGAGCTGGCATACGA
CCL5	CCAGCAGTCGTCTTTGTCAC	CTCTGGGTTGGCACACACTT
IL6	CCCACCGGGAACGAAAGAG	GGACCGAAGGCGCTTGT
ACTB (β-actin)	GTTGTCGACGACGAGCG	GCACAGAGCCTCGCCTT
Q-PCR primers (5’ to 3’) for mouse genes:		
Ccl2	CACTCACCTGCTGCTACTCA	GCTTGGTGACAAAAACTACAGC
Ccl5	GCTGCTTTGCCTACCTCTCC	TCGAGTGACAAACACGACTGC
Cxcl5	CACTCGCAGTGGAAAGAACG	CGTGGGTGGAGAGAATCAGC
Cxcl1	ACTCAAGAATGGTCGCGAGG	ACTTGGGGACACCTTTTAGCA
Cx3cl1	GCGACAAGATGACCTCACGA	TGTCGTCTCCAGGACAATGG
Cxcl11	TGGAACATGCAGCCACGTAT	CCACAGAAGGTAGCGTGGAG
Ccl4	CTGTGCAAACCTAACCCCGA	AGGGTCAGAGCCCATTGGT
Ccl7	CCCTGGGAAGCTGTTATCTTCAA	CTCGACCCACTTCTGATGGG
Cxcl3	GAAAGGAGGAAGCCCCTCAC	ACACATCCAGACACCGTTGG
Cxcl16	AATTGGCTGGATGTCGGCTA	AACGCAAGAGACAAGGGTCC
Cxcl10	TGAATCCGGAATCTAAGACCATCAA	AGGACTAGCCATCCACTGGGTAAAG
Il12b	CGCCACACAAATGGATGCAA	TGTGTCCTGAGGTAGCCGTA
Ifna2	ATGAGGAGGCTCCCCTTTCTC	AGTAAGATCTCGCAGCACAGG
Ifnb1	CCCTATGGAGATGACGGAGA	CCCAGTGCTGGAGAAATTGT
Il12a	CAAGAGACACAGTCCTGGGAA	GCTGACCTTGGGAGACACAT
Il6	GAGGATACCACTCCCAACAGACC	AAGTGCATCATCGTTGTTCATACA

Il16	TTAGTCCAATCAGGGCGTGG	CACTGCATGAGTCCCTGACC
Il1a	CCCATGATCTGGAAGAGACCA	CAAACTTCTGCCTGACGAGC
Ifng	ACAGCAAGGCGAAAAAGGATG	TGGTGGACCACTCGGATGA
Il7	CGCAGACCATGTTCCATGTTTC	TGTGACAGGCAGCAGAACAA
Il24	TTTAGGACCCTAGCAGGAGCA	TTGGCAAGACCCAAATCGGA
Actb (β-actin)	GGCTGTATTCCCCTCCATCG	CCAGTTGGTAACAATGCCATGT

Supplementary Table 2. The Q-PCR primers used in this study.

The human cancer data (Supplementary Fig. 1a) were derived from TCGA (version 2016-09-01) databases[1].
The human cancer data (Supplementary Fig. 8a) were derived from databases[2]: [Melanoma (on-treatment anti-PD-1 (GSE91061, GSE115821); Melanoma (on-treatment anti-CTLA-4 (GSE115821).
The human cancer data (Supplementary Fig. 8b) were derived from databases[3]: Anti-CTLA-4 treatment: Melanoma (GIDE2019, GSE165278, LIU2019, VANALLEN2015); Anti-PD-1 plus CTLA-4 treatment: Melanoma (GIDE2019, LIU2019); Anti-PD-L1 treatment: Esophageal adenocarcinoma (GSE165252, GSE183924).
The human cancer data (Supplementary Fig. 8c) were derived from databases[3]: Anti-CTLA-4 treatment: Melanoma (GIDE2019, LIU2019, VANALLEN2015); Anti-PD-L1 treatment: Esophageal adenocarcinoma (GSE165252, GSE183924).
The human cancer data (Supplementary Fig. 12a) were derived from the GTEx (version no. 7—15 May 2019), TCGA and TARGET (version no. 15.0—20 February 2019) databases[4].
The human cancer data (Supplementary Fig. 17) were derived from TCGA biolinks[5].

Supplementary Table 3. The human cancer data were obtained from the corresponding databases.

REFERENCES

1. Li, C., Tang, Z., Zhang, W., Ye, Z. & Liu, F. A.-O. GEPIA2021: integrating multiple deconvolution-based analysis into GEPIA. *Nucleic. Acids. Res* **49**, W242-W246 (2021).
2. Kovács, S. A., Fekete, J. T. & Győrffy, B. Predictive biomarkers of immunotherapy response with pharmacological applications in solid tumors. *Acta. Pharmacol. Sin* (2023).
3. Kovács, S. A. & Győrffy, B. Transcriptomic datasets of cancer patients treated with immune-checkpoint inhibitors: a systematic review. *J. Transl. Med* **20**, 249 (2022).
4. Bartha, Á. & Győrffy, B. TNMplot.com: A Web Tool for the Comparison of Gene Expression in Normal, Tumor and Metastatic Tissues. *Int. J. Mol. Sci* **22**, 2622 (2021).
5. Colaprico, A. *et al.* TCGAbiolinks: an R/Bioconductor package for integrative analysis of TCGA data. *Nucleic. Acids. Res* **44**, e71 (2016).
