# Supplementary material for: ARIH1 activates STING-mediated T-cell activation and sensitizes tumors to immune checkpoint blockade
Source: Nat Commun. 2023 Jul 10;14:4066. doi: 10.1038/s41467-023-39920-5 (PMC10333294; doi:10.1038/s41467-023-39920-5)
Supplement: Supplementary file 3 — Reporting Summary [file 41467_2023_39920_MOESM3_ESM.pdf]

nature portfolio

Corresponding author(s): Hongguang Xia

Last updated by author(s): Jun 15, 2023

Reporting Summary

Nature Portfolio wishes to improve the reproducibility of the work that we publish. This form provides structure for consistency and transparency in reporting. For further information on Nature Portfolio policies, see our Editorial Policies and the Editorial Policy Checklist.

Statistics

For all statistical analyses, confirm that the following items are present in the figure legend, table legend, main text, or Methods section.

n/a	Confirmed	
☐	☒	The exact sample size (n) for each experimental group/condition, given as a discrete number and unit of measurement
☐	☒	A statement on whether measurements were taken from distinct samples or whether the same sample was measured repeatedly
☐	☒	The statistical test(s) used AND whether they are one- or two-sided<br>*Only common tests should be described solely by name; describe more complex techniques in the Methods section.*
☒	☐	A description of all covariates tested
☒	☐	A description of any assumptions or corrections, such as tests of normality and adjustment for multiple comparisons
☐	☒	A full description of the statistical parameters including central tendency (e.g. means) or other basic estimates (e.g. regression coefficient) AND variation (e.g. standard deviation) or associated estimates of uncertainty (e.g. confidence intervals)
☐	☒	For null hypothesis testing, the test statistic (e.g. F, t, r) with confidence intervals, effect sizes, degrees of freedom and P value noted<br>*Give P values as exact values whenever suitable.*
☒	☐	For Bayesian analysis, information on the choice of priors and Markov chain Monte Carlo settings
☒	☐	For hierarchical and complex designs, identification of the appropriate level for tests and full reporting of outcomes
☒	☐	Estimates of effect sizes (e.g. Cohen's d, Pearson's r), indicating how they were calculated

Our web collection on statistics for biologists contains articles on many of the points above.

Software and code

Policy information about availability of computer code

Data collection	Flow cytometry data were collected using CytExpert v2.4 (Beckman Coulter).<br>Imaging data were collected using Zeiss LSM 880 with AiryScan and ScanScope CS2.<br>Cell proliferation data were collected using Varioskan LUX.
Data analysis	GraphPad Prism 8.0 was used for statistical analyses.<br>ZEN 2 (blue edition) and ImageJ 1.53a were used for immunofluorescence images processing.<br>ImageScope was used for immunohistochemistry images processing.<br>CytExpert v2.4 was used for the analysis and quantification of flow cytometry.<br>The R package Cluster Profiler (version 4.2.2) was used for performing Gene Set Enrichment Analysis (GSEA) on RNA sequencing data.<br>MaxQuant v1.5 was used for protein identification and quantification in mass spectrometry data.

For manuscripts utilizing custom algorithms or software that are central to the research but not yet described in published literature, software must be made available to editors and reviewers. We strongly encourage code deposition in a community repository (e.g. GitHub). See the Nature Portfolio guidelines for submitting code & software for further information.

Data

Policy information about availability of data

All manuscripts must include a data availability statement. This statement should provide the following information, where applicable:

- Accession codes, unique identifiers, or web links for publicly available datasets
- A description of any restrictions on data availability
- For clinical datasets or third party data, please ensure that the statement adheres to our policy

Data Availability statement
The mass spectrometry proteomics data generated in this study have been deposited to the ProteomeXchange Consortium via the iProX partner repository with the dataset identifier PXD041429 (https://proteomecentral.proteomexchange.org/cgi/GetDataset?ID=PXD041429). The RNA-seq data from Arih1-WT-OE and Arih1-C355S-OE 4T1 cells generated in this study have been deposited in the Gene Expression Omnibus (GEO) database under the accession numbers GSE231726 (https://www.ncbi.nlm.nih.gov/geo/query/acc.cgi?acc=GSE231726). The human cancer data (Supplementary Fig. 1a, Supplementary Fig. 8a-c, Supplementary Fig. 12a, and Supplementary Fig. 17) were derived from databases listed in Supplementary Table 3. The remaining data are available within the article, supplementary information, and source data file. Source data are provided with the paper.

Research involving human participants, their data, or biological material

Policy information about studies with human participants or human data. See also policy information about sex, gender (identity/presentation), and sexual orientation and race, ethnicity and racism.

Reporting on sex and gender	No sex-and gender-based analyses have been performed. Tumor patients were all female, not gender-related.
Reporting on race, ethnicity, or other socially relevant groupings	No race-, ethnicity-, and other socially relevant groupings-based analyses have been performed. All patients recruited in this study were Asian (Chinese) females, from Taizhou Hospital of Zhejiang Province affiliated to Wenzhou Medical University.
Population characteristics	Tumors from triple-negative breast cancer (TNBC) and Normal tissues were obtained from 6 female patients (6 cases each group, median age: 51 years old, range from 43 to 59).
Recruitment	All cancer patients volunteers were from Taizhou Hospital of Zhejiang Province affiliated to Wenzhou Medical University, following ethical guidelines. All patients willingly provided their samples at no cost after signing the informed consent form. The selection of cancer patients was random, and no biases related to sex or gender were performed.
Ethics oversight	The study was approved by the Taizhou Hospital of Zhejiang Province affiliated to Wenzhou Medical University Ethics Committee.

Note that full information on the approval of the study protocol must also be provided in the manuscript.

Field-specific reporting

Please select the one below that is the best fit for your research. If you are not sure, read the appropriate sections before making your selection.

☒ Life sciences ☐ Behavioural & social sciences ☐ Ecological, evolutionary & environmental sciences

For a reference copy of the document with all sections, see nature.com/documents/nr-reporting-summary-flat.pdf

Life sciences study design

All studies must disclose on these points even when the disclosure is negative.

Sample size	Sample size estimates has been performed on previous experience to obtain statistical significance and reproducibility. For in vitro experiments such as Western blot, qPCR, IHC, IF and flow cytometry, at least three samples were used per group for minimal statistics requirements. For in vivo studies, the sample size was determined to be sufficient to obtain the statistical difference between groups.
Data exclusions	There are no data exclusions.
Replication	All experiments underlying main conclusions of this study have been successfully replicated multiple times and corroborated by several models. All the western blot, qPCR, IHC, IF and flow cytometry were carried out at least three independent times with the same results.
Randomization	Samples and organisms were randomly allocated to experimental groups. No specific randomization protocol has been used. Mice were age- and sex matched.
Blinding	No specific blinding was applied since all experiments were assigned into groups including relevalant controls and analysis was done objectively and without bias.

Reporting for specific materials, systems and methods

We require information from authors about some types of materials, experimental systems and methods used in many studies. Here, indicate whether each material, system or method listed is relevant to your study. If you are not sure if a list item applies to your research, read the appropriate section before selecting a response.

Materials & experimental systems

n/a	Involved in the study
☐	☒ Antibodies
☐	☒ Eukaryotic cell lines
☒	☐ Palaeontology and archaeology
☐	☒ Animals and other organisms
☒	☐ Clinical data
☒	☐ Dual use research of concern
☒	☐ Plants

Methods

n/a	Involved in the study
☒	☐ ChIP-seq
☐	☒ Flow cytometry
☒	☐ MRI-based neuroimaging

Antibodies

Antibodies used

The antibodies were provided as follows: DNA-PKcs (#ET1610-12, 1 : 2000, HUABIO), DNA-PKcs (#sc-5282, 1:200, Santa Cruz Biotechnology), TBK1 (#3504, 1 : 1000, Cell Signaling Technology), p-TBK1 (Ser172) (#AP1026, 1:1000, ABclonal), IRF3 (#A0816, 1:100, ABclonal), p-IRF3 (Ser396) (#29047, 1:100, Cell Signaling Technology), STING (#13647, 1:1000, Cell Signaling Technology), STING (#ET1705-68, 1:1000, HUABIO), p-STING (Ser366) (#19781, 1:1000, Cell Signaling Technology), p-STING (Ser366) (#AP1223, 1:1000, ABclonal), cGAS (#HA500023, 1 : 1000, HUABIO), Lamp2 (#sc-18822, 1:200, Santa Cruz Biotechnology), dsDNA (#sc-58749, 1:200, Santa Cruz Biotechnology), ARIH1 (C-7) (#sc-514551, 1 : 50; Santa Cruz Biotechnology), ARIH1 (Goat) (#EB05812, 1:1000, Everestbiotech), GST (B-14) (#sc-138, 1 : 200, Santa Cruz Biotechnology), γ-H2AX (#ET1602-2, 1 : 1000, HUABIO), H2AX (#ET1705-97, 1 : 1000, HUABIO), 53BP1 (#ET1704-05, 1 : 1000, HUABIO), Ubiquitin (P4D1) (#sc-8017, 1: 200, Santa Cruz Biotechnology), His-tag (#66005-1-Ig, 1 : 1000, Proteintech), Flag-tag (#0912-1, 1 : 2000, HUABIO), HA-tag (#0906-1, 1: 2000, HUABIO), Tubulin (#M1305-2, 1:5000, HUABIO) and β-Actin (#M1210-2, 1 : 2000, HUABIO). The antibodies for immunohistochemistry (IHC) were used: ARIH1 (#EB05812, 1:100, Everest biotech), CD8α (#98941, 1:200, Cell Signaling Technology), CD8α (#ab17147, 1:100, Abcam), GzmB (#44153, 1:100, Cell Signaling Technology), IRF3 (#A11118, 1:100, HUABIO), DNA-PKcs (#ET1610-12, 1:200, HUABIO). The following antibodies for flow analysis were displayed: Zombie Violet™ Fixable Viability Kit (#423114; 1:200; Biolegend), PerCP/Cyanine5.5 anti-mouse CD45 (#103132; 1:200; Biolegend), PE/Cyanine7 anti-mouse CD3 (#100320; 1:200; Biolegend), FITC anti-mouse CD8 (#100706; 1:200; Biolegend), APC anti-human/mouse Granzyme B (#372204; 1: 200; Biolegend). The secondary antibodies for western blot were used: goat anti-mouse(#31430,1:20000, Thermo Fisher Scientific), goat anti-rabbit (#31460, 1:20000, Thermo Fisher Scientific), donkey anti-goat (#A0181, 1:1000, Beyotime). The fluorescent secondary antibodies for immunofluorescence were used: goat anti-rabbit Alexa Fluor 555 (#A-21428, 1:500, Thermo Fisher Scientific), goat anti-mouse DyLight 649 (#A23610, 1:500, Abbkine). The in vivo antibodies for mouse models were used: control antibody InVivoMab rat IgG2b isotype (#BE0090; 100 or 200μg; Bioxcell), anti-PD-L1 (#BE0101; 100 or 200μg; Bioxcell).

Validation

For all the antibodies, we carried out western blot according to the method on the company's website, and detected whether the band size met the expectation with molecular weight marker, and added appropriate positive control and negative control. For human ARIH1 and cGAS, we added siRNA knockdown verification. For murine Arih1 and Sting, we added shRNA knockdown verification. Antibodies purchased from Cell Signaling Technology were validated as per their website stating "Antibody signal is measured in model systems with known presence/absence of target signal. Includes wild-type vs. genetic knockout, targeted induction or silencing." Besides, each antibody's manual contains authentic data results from the companies (HUABIO, ABclonal, Cell Signaling Technology, and Santa Cruz Biotechnology) validating specificity, and our data also verifies the corresponding antibody's specificity.

Eukaryotic cell lines

Policy information about cell lines and Sex and Gender in Research

Cell line source(s)

HEK293T (ATCC CRL-3216), HeLa (ATCC CRM-CCL-2), U2OS (ATCC HTB-96), MCF-7 (ATCC HTB-22), MDA-MB-231 (ATCC CRM-HTB-26), 4T1 (ATCC CRL-2539), E0771 (ATCC CRL-3405), B16-F10 (ATCC CRL-6475), LLC (ATCC CRL-1642), MC38 (Kerafast ENH204-FP), and CT26 (ATCC CRL-2638) cell lines were obtained from the American Type Culture Collection (ATCC) and Kerafast, Inc.. 4T1-Arih1-WT-OE, 4T1-Arih1-C355S-OE, 4T1-Arih1-WT-OE&Sting-KD, 4T1-Arih1-KD, 4T1-Sting-KD, E0771-Arih1-WT-OE, E0771-Arih1-C355S-OE, B16-F10-Arih1-WT-OE, and B16-F10-Arih1-C355S-OE cells were generated by our laboratory through lentiviral transduction.

Authentication

All cell lines were authenticated by providers (STR profiling).

Mycoplasma contamination

All used cell lines were tested negative for contamination.

Commonly misidentified lines
(See ICLAC register)

None of commonly misidentified cell lines has been used.

Animals and other research organisms

Policy information about studies involving animals; ARRIVE guidelines recommended for reporting animal research, and Sex and Gender in Research

Laboratory animals	Female BALB/c mice, C57BL/6 mice and nude mice, and male C57BL/6 mice (aged 6-8 weeks) were purchased from Shanghai SLAC Laboratory Animal Co., Ltd. (Shanghai, China). All the animal experiments were strictly conducted in accordance with the protocols approved by the Ethics Committee for Animal Studies at Zhejiang University, China. All mice were cultured in suitable temperature and humidity environment (25°C, suitable humidity (typically 50%) ,12 hour dark/light cycle), and fed with sufficient water and food .
Wild animals	We don't use wild animals.
Reporting on sex	We used female BALB/c , C57BL/6 and nude mice. We also used male C57BL/6 mice. We used 4T1 and E0771 cells in female mice to mimic human triple-negative breast cancer (TNBC). 4T1 and E0771 cells can be transplanted into the fat pad of the mouse mammary gland, in contrast to male mice, these tumor cells are highly tumorigenic, invasive, and spontaneously metastatic. Experiments with 4T1 and E0771 cells in female mice could provide a valuable model system for preclinical TNBC studies. For the tumor metastasis model involving the B16-F10 cell line, we selected male mice according to a previous study that male mice are more suitable for studying melanoma tumor models within the immune microenvironment [PMID: 32235862].
Field-collected samples	no field collected samples were used in the study.
Ethics oversight	All mice were kept in a specific pathogen-free (SPF) facility. Carbon dioxide was used for euthanasia. Mice were sacrificed when tumor volumes exceeded 2000mm3 or when tumors reached over 20 mm in any dimension. All the animal experiments were strictly conducted in accordance with the protocols approved by the Tab of Animal Experimental Ethical Inspection of the First Affiliated Hospital, College of Medicine, Zhejiang University (Reference Number: (2022) Real Action Fast Review No. (847)).

Note that full information on the approval of the study protocol must also be provided in the manuscript.

Flow Cytometry

Plots

Confirm that:

- [x] The axis labels state the marker and fluorochrome used (e.g. CD4-FITC).
- [x] The axis scales are clearly visible. Include numbers along axes only for bottom left plot of group (a 'group' is an analysis of identical markers).
- [x] All plots are contour plots with outliers or pseudocolor plots.
- [x] A numerical value for number of cells or percentage (with statistics) is provided.

Methodology

Sample preparation	Tumors were collected and processed into single-cell suspensions through digestion in collagenase type I (#2350118, Gibco) and Dnase I (#143582, Roche) at 37°C for 45 min. After filtering with a 45 μm filter (BD Bioscience), the isolated cells were stained with the specific surface marker antibodies, anti-CD45-Percp-Cy5.5 (#103132; Biolegend), anti-CD3-PE-Cy7(#100320; Biolegend) and anti-CD8-FITC (#100706; Biolegend) in PBS for 30 min at 4°C. Intracellular staining of GzmB was performed as follows: cells were washed and then fixed and permeabilized with a Fix/Perm kit (#421403; Biolegend), and finally stained with anti-APC-GzmB (#372204; Biolegend). For proper compensation of flow cytometry channels, single-stain samples were utilized.
Instrument	CytoFlex analyzer (Beckman Coulter)
Software	CytExpert v2.4 and GraphPad Prism 8.0
Cell population abundance	moderate
Gating strategy	In our experiment, zombie was used to gate the living cells, Percp-Cy5.5 conjugated-CD45 antibody was used to gate the immune cells, PE-Cy7 conjugated-CD3 antibody was used to gate the T cells, FITC-conjugated-CD8 antibody was used to circle the cytotoxic T cells, and APC conjugated-GzmB antibody was used to gate the activated cytotoxic T cells.

- [x] Tick this box to confirm that a figure exemplifying the gating strategy is provided in the Supplementary Information.
